# Quorum sensing-mediated inter-specific conidial anastomosis tube fusion between *Colletotrichum gloeosporioides* and *C. siamense*

**DOI:** 10.1186/s43008-021-00058-y

**Published:** 2021-04-01

**Authors:** Nikita Mehta, Abhishek Baghela

**Affiliations:** 1 National Fungal Culture Collection of India (NFCCI), Biodiversity and Palaeobiology Group, MACS-Agharkar Research Institute, G.G. Agarkar Road, Pune, 411004 India; 2 Savitribai Phule Pune University, Pune, 411007 India

**Keywords:** Conidial anastomosis tube, Heterokaryosis, Quorum sensing, Genetic diversity, Hybrid vigour, *Colletotrichum*

## Abstract

Many plant pathogenic filamentous fungi undergo fusion of conidia through conidial anastomosis tubes (CATs), which is believed to facilitate horizontal gene transfer between species. We discovered a remarkable inter-specific CAT fusion between two important plant fungal pathogens *Colletotrichum gloeosporioides* and *C. siamense*. In an invitro assay, under no selection pressure, the inter-specific CAT fusion was preferred with higher frequency (25% ± 5%) than intra-specific CAT fusion (11% ± 3.6%). Different stages of CAT fusion viz. CAT induction, homing, and fusion were observed during this inter-specific CAT fusion. The CAT fusion was found to be higher in absence of nutrients and under physiological stresses. This CAT fusion involved a quorum sensing phenomenon, wherein the CAT induction was dependent on conidial density and the putative quorum sensing molecule was extractable in chloroform. Movement of nuclei, mitochondria, and lipid droplets were observed during the CAT fusion. Post CAT fusion, the resulting conidia gave rise to putative heterokaryotic progenies with variable colony characteristics as compared to their parental strains. Few heterokaryons showed variable AFLP banding pattern compared to their parental strains, thereby suggesting a possible genetic exchange between the two species through CAT fusion. The heterokaryotic progenies exhibited varied fitness under different stress conditions. Our study illustrated a possible role of inter-specific CAT fusion in generation of genetic and phenotypic diversity in these fungal pathogens.

**Supplementary Information:**

The online version contains supplementary material available at 10.1186/s43008-021-00058-y.

## INTRODUCTION

*Colletotrichum* is a widespread genus voted as the eighth most important group of plant pathogenic fungi in the world (Dean et al. 2012). Among different species, *C. gloeosporioides* and *C. siamense* cause infection in a wide range of host plants (Cai et al. 2009; Freeman et al. 1996; Hyde et al. 2009; Jayawardena 2018; Sharma et al. 2013). We have previously reported a high level of genetic diversity in *C. gloeosporioides* isolates in India (Mehta et al. 2017). Our observation also corroborates with earlier studies which discussed genetic and pathogenic diversity in *C. gloeosporioides* (Denoyes-Rothan et al. 2003; Nova et al. 2011; Weeds et al. 2003). The possible mechanism that gives rise to such high genetic diversity may lie in meiotic recombination during sexual reproduction, however, it has been suggested that sexual reproduction in *C. gloeosporioides* in nature would be rare, if not absent (da Silva et al. 2020; Weir et al. 2012). Similarly, sexual stage for *C. siamense* has not been demonstrated to date (Prihastuti et al. 2009).

Horizontal gene transfer (HGT) or chromosome transfer (HCT) may contribute to the high level of genetic variation observed in fungi that lack sexual reproduction (Ishikawa et al. 2012; Mehrabi et al. 2011). HCT and HGT have been considered to play an important role in the evolution of plant fungal pathogens (Rosewich and Kistler 2000; Walton 2000; Oliver and Solomon 2008; Rep and Kistler 2010; Mehrabi et al. 2011). HCT has been demonstrated in *Alternaria alternata*, *Fusarium oxysporum* and *C. gloeosporioides* under laboratory conditions in-vitro (Akagi et al. 2009; He et al. 1998; Ma et al. 2010; Manners and He 2011). The cellular basis of HGT/HCT is ambiguous in general, and especially in case of *Colletotrichum* species.

Typically, a conidium develops to form a germ tube (GT) that extends and successively branches to establish the fungal colony. However, under certain conditions, the conidia and conidial germlings in close vicinity to each other may form specialized structures, called conidial anastomosis tubes (CATs), resulting in an interconnected germling network (Read et al. 2010; Roca et al. 2003, 2005a, b). Such system can function as a coordinated network which regulates its overall homeostasis by sharing nutrients, water, signal molecules and organelles, while allowing genetic exchange among conidia (Read et al. 2009). CAT fusion seems to involve quorum sensing (QS), wherein, an individual conidium discern their density by detecting the extracellular quorum sensing molecules (QSMs) secreted by the conidial population, which prime conidia for CAT fusion (Roca et al. 2005a). An unknown CAT inducer in *Neurospora crassa* appears to activate a mitogen-activated protein (MAP) kinase, which is shown to be vital for CAT fusion (Roca et al. 2005a). *N. crassa* has three different MAPK pathways: Cell Wall Integrity (CWI), MAK-2, and Osmotic Stress (OS). Both the CWI and MAK-2 pathways are activated by CAT fusion signalling. It has been reported that two proteins namely a MAP kinase (MAK-2) and SO direct cell fusion (Fischer et al. 2018; Fischer and Glass 2019; Fleissner et al. 2009; Palma-Guerrero et al. 2009). However, the chemical nature of QSM/inducer responsible for CAT fusion has not been identified for any fungus to date. Since CAT fusion involves transfer of genetic material, it is hypothesized that CAT fusion may account for HGT and/or HCT between otherwise incompatible strains (Ma et al. 2010; Mehrabi et al. 2011).

CAT fusion has been demonstrated in many species of fungi (Roca et al. 2005b). However, CAT fusion has been extensively studied in *C. lindemuthianum, C. gossypii, C. fructicola, C. nymphaeae, F. oxysporum, N. crassa, Epichloë festucae* and *Venturia inaequalis* and for many of these fungi; in-vitro methods have been developed to examine the biology of CAT fusion (Gonçalves et al. 2016; Kurian et al. 2018; Leu 1967; Roca et al. 2003, 2005a; Shahi et al. 2016; Tanaka et al. 2013, 2020; Wu et al. 2014). It has been suggested that CAT fusion might play a vital role in the life cycle of fungi by improving the chances of colony establishment during nutrient starvation (Roca et al. 2005b); however, there are some disparity in relationship between CAT induction and nutrient availability. In *C. lindemuthianum*, CAT fusion does not occur in PDB and even in nutrient poor Vogel’s medium, while it occurs only in water (Ishikawa et al. 2010). In other CAT forming fungi like *V. inaequalis* and *F. oxysporum*, CAT fusion does not occur in water, while it requires some amount of nutrients for CAT fusion e.g. complete medium for *V. inaequalis* and YNB + KNO_3_ /1% PDB + NaNO_3_ for *F. oxysporum* (Glass and Fleissner 2006; Kurian et al. 2018; Leu 1967; Shahi et al. 2016). The possibility of CAT fusion as a consequence of physiological stresses has also not been assessed.

CAT fusion has been studied in multiple species of *Colletotrichum* viz. *C. lindemuthianum, C. gossypii, C. fructicola,* and *C. nymphaeae* (Gonçalves et al. 2016; Ishikawa et al. 2010; Roca et al. 2003, 2004). Two economically important species of this genus i.e., *C. gloeosporioides* and *C. siamense* are the members of *Colletotrichum gloeosporioides* species complex (Weir et al. 2012). Out of these two species, formation of multiple appressoria and CATs in *C. gloeosporioides* was reported on apple leaves (Araujo and Stadnik 2013). However, no efforts were undertaken to analyze the CAT fusion dynamics in-vitro and deduce the physiological requirements for CAT fusion in *C. gloeosporioides* and *C. siamense.* Further, whether CAT fusion contributes to generation of phenotypic and genetic diversity in *C. gloeosporioides* and *C. siamense* remains unexplored.

Various inter-specific hybrids of filamentous plant pathogens have been described (Depotter et al. 2016) so far and CAT fusion has been implicated for generation of fungal hybrids. In one such study, an interspecific hybrid of *C. lindemuthianum* and *C. gossypii* was reported to occur through CAT fusion and the hybrids exhibited morphological and genetic traits of both the parent species (Roca et al. 2004). Though the existence of hybrids in *C. gloeosporioides* species complex was predicted in early studies (Cisar et al. 1994), evidence of any inter-specific hybrids in this species complex is yet to be demonstrated. Inter-specific gene transfer or hybridization between closely related species is a fast means of genome evolution, which allows organisms to acquire novel traits to colonize new niches and/or novel hosts (Man In’T Veld et al. 2007; Bertier et al. 2013). Furthermore, it is speculated that hybrid pathogens may possess fitness advantages or disadvantages in a given niche (Samarasinghe et al. 2020; Stukenbrock 2016).

In the present work, physiological requirements and dynamics of CAT fusion between *C. gloeosporioides* and *C. siamense* were determined. We elucidated that CAT fusion is mediated through a quorum sensing like phenomenon in these fungi. Experimental proofs were generated to support the role of CAT fusion in generating phenotypic and genotypic diversity in these fungi. The putative heterokaryons of *C. gloeosporioides* exhibited variable fitness under different stress conditions. Taken together, our data demonstrate how a chloroform-extractable signalling molecule mediates CAT fusion, which ultimately influences genetic and phenotypic diversity in these fungal pathogens.

## MATERIALS AND METHODS

### Fungal strains and species confirmation by *ApMAT* sequencing and phylogeny

*Colletotrichum gloeosporioides* (CBS 953.97) and *C. siamense* (NFCCI 3061) strains used in the study were obtained from Microbial Type Culture Collection (MTCC), Chandigarh, India and National Fungal Culture Collection of India (NFCCI) (WDCM-932), Agharkar Research Institute, Pune, India respectively. The strains were maintained on Potato Dextrose Agar (PDA) (Hi-Media Laboratories Pvt. Ltd.) plates at 25 °C and preserved in 15% glycerol at − 80 °C. The species identities of the isolates were initially confirmed by microscopic morphology and further the molecular identification of the above strains was done by *ApMAT* gene sequencing. Briefly, the genomic DNA (gDNA) was isolated from fungal colonies grown on PDA  plates for a week, by following a rapid DNA extraction protocol (Aamir et al. 2015) using FastPrep®24 tissue homogenizer (MP Biomedicals GmbH, Germany). The gDNA were subjected to polymerase chain reaction (PCR) amplification of the *ApMAT* gene using primers AMF - TCATTCTACGTATGTGCCCG and AMR - CCAGAAATACACCGAACTTGC using standard cycling conditions as described previously (Silva et al. 2012). The PCR products were purified using QIAquick PCR Purification Kit (QIAGEN), and sequenced using Big Dye Terminator cycle sequencing kit (Applied Biosystems, Foster City, CA) as per the manufacturer’s instructions on an ABI 3100 Avant Prism automated DNA sequencer (Applied Biosystems). The sequences generated in this study were deposited in NCBI-GenBank. The *ApMAT* region sequences of closely related species of *C. gloeosporioides* and *C. siamense* were retrieved from NCBI-GenBank and a phylogenetic tree was constructed using these sequences by following neighbor-joining method in MEGA7 to confirm the phylogenetic positions of *C. gloeosporioides* and *C. siamense* strains used in the present study.

### In-vitro conidial anastomosis tube induction and their dynamics

The *C. gloeosporioides* and *C. siamense* strains were inoculated on bean pod agar medium (autoclaved French bean pods submerged in 2% water agar) and were incubated in the dark at 25 °C to induce sporulation. Post-inoculation the conidia were harvested from these two species individually at different time points viz. 6, 10, 13, 17 and 20 days and suspended in distilled water. In order to induce CAT fusion, 0.4 ml conidial suspension (1 × 10^6^ per ml) and 0.6 ml distilled water were placed in the wells of a 24-well tissue culture plate (Tarsons, India) with the final concentration of conidia being 4 × 10^5^/ml and incubated for 24 h, 48 h, 72 h and 96 h in the dark at 25 °C. The CAT induction was carried out in three different ways; 1) CAT induction in only *C. gloeosporioides* conidia, 2) CAT induction in only *C. siamense* conidia, and 3) CAT induction in a co-culture of *C. gloeosporioides* and *C. siamense* conidia in equal numbers (Fig. S1). Different stages of CAT like induction, homing and fusion were examined at different time points using an Olympus BX53 DIC microscope equipped with Olympus DP73 camera and CellSens 1.13 imaging software. This experiment was performed in triplicates and the CAT fusion was quantified as the percentage of conidia involved in fusion (Roca et al. 2003). A total of 150 conidial pairs were counted per experimental replicate.

To study whether GT formation and CAT fusion occurs concomitantly or they are mutually exclusive, *C. gloeosporioides* and *C. siamense* were grown on bean pod agar and different aged conidia (6, 10, 13 and 17) at a final concentration 4 × 10^5^ per ml were co-cultured in distilled water (1 ml) and 100% PDB (1 ml) in a 24-well tissue culture plate and incubated for 18 h for germ tube GT formation and 72 h for CAT fusion in dark at 25 °C. GT formation and CAT fusion were then examined under inverted microscope (Olympus BX53 with Olympus DP73 camera, Cellsens 1.13 imaging software) using differential interference contrast (DIC) optics.

### Effects of nutrients, physiological stresses and known CAT inducers on CAT induction

In order to study whether CAT fusion between *C. gloeosporioides* and *C. siamense* is dependent on nutrient availability and/or physiological stress conditions, the CAT fusion frequency was determined in the presence of different stresses and nutrients. The CAT fusion in 17 days old conidia of both the strains (co-culture) were induced in water, 100% Potato dextrose broth (PDB), 30 mM H_2_O_2_ (oxidative stress), 1 M NaCl and 1 M Sorbitol (osmotic stress), at 40 °C (heat stress) and 100 μg/ml Copperoxychloride (antifungal stress). Further, the CAT fusion in 17 days old conidia of both the strains (co-culture) were also tested in the presence of various carbon and nitrogen compounds like 2% Glucose, 100 mM KNO_3_, combination of 2% glucose and 100 mM KNO_3_ (Shahi et al. 2016). The CAT induction was also assessed in the presence of previously known CAT inducers of other fungal systems e.g. 25 mM NaNO_3_, and 25 mM MgCl_2_ (Kurian et al. 2018). Effect of a known CAT inhibitor tryptophan (50 μM) (Fischer-Harman et al. 2012) on CAT induction in these fungi was also assessed. To study whether the CAT induction in these two species involves a MAP kinase kinase (MEK), the CAT fusion percentage was also determined in the presence of a MEK inhibitor InSolution™ PD98059 (5 μM).

### Detection of quorum sensing like phenomenon during CAT fusion

Different numbers of conidia were tested to find out the threshold of conidial numbers required for inter-specific CAT fusion between *C. gloeosporioides* and *C. siamense.* The numbers of conidia tested were 4 × 10^2^, 4 × 10^3^, 4 × 10^4^, 4 × 10^5^ and 4 × 10^6^ per ml of water. The conidial numbers represent sum of equal concentration of *C. gloeosporioides* and *C. siamense* conidia.

To detect the secretion of unknown QSMs during CAT fusion, post inter-specific CAT fusion (96 h of incubation) of the 17 days old conidia of both the species, the conidial suspension was collected from the tissue culture plate and conidia were separated from the liquid medium (water) by centrifugation at 10,000 rpm for 10 min at RT. The obtained CAT medium supernatant was filtered through Whatman® Syringe filter (pore size 0.45 μm). This CAT medium supernatant (1 ml) was tested for their ability to induce CAT fusion in young (6 days) as well as old conidia (17 days) of *C. gloeosporioides* and *C. siamense* in co-culture (4 × 10^5^ conidial concentration), wherein, the conidial co-culture in water (1 ml) was used as a control. These conidial co-cultures were incubated at 25 °C for 72 h in the dark. The CAT induction ability of the CAT medium supernatant was tested even in the presence of nutrients viz. 100% PDB by inoculating 6 and 17 days old conidia of both the strains in (a) 1 ml CAT medium supernatant, (b) 1 ml 100% PDB individually and (c) combination of equal volume of CAT medium supernatant and PDB and (d) combination of equal volume of PDB and water.

To understand the basic chemical nature of putative QSM, three different sets of experiments were performed viz. 1) the CAT medium supernatant (1 ml) was treated with equal volume of chloroform and an aqueous suspension was used to induce CAT fusion, 2) the leftover chloroform (1 ml) from the first set of experiment was treated with water (1 ml) to re-extract the water soluble QSMs, if any, 3) the CAT medium supernatant (1 ml) was also treated with 4 μl of proteinase K (10 mg/ml) for 3 h at 55 °C. Such treated CAT medium supernatants were tested for their ability to induce CAT fusion. The control experiments included CAT fusion in 1) water, 2) combination of equal volume of PDB and water, 3) combination of equal volume of PDB and chloroform treated CAT medium supernatant, 4) combination of equal volume of PDB and proteinase K treated CAT medium supernatant, and 5) combination of equal volume of PDB and water re-extract.

### Nuclear and organelles transfer during CAT fusion

To visualize the genetic transfer between *C. gloeosporioides* and *C. siamense* during CAT fusion, DAPI staining was performed with some modifications (James et al. 1995; Roca et al. 2003). The nuclear staining was observed using fluorescence microscopy (ZIESS AXIO Imager.A2 microscope with AxioCam MRc5 camera). Few additional organelles staining experiments were performed using 100 nM MitoRed (Sigma-Aldrich, USA), and 100 nM Nile Red (Sigma-Aldrich, USA) (Hickey et al. 2004; Meadows 2012) to visualize the transfer of mitochondria, and lipid droplets, respectively during the CAT fusion. Confocal microscopy was performed to visualize the migration of above-mentioned organelles using Leica SP8 confocal microscope and images were processed and analyzed by LAS X software.

### Assessment of colony morphology of progenies generated through intra and inter-specific CAT fusion

Post intra-specific (self-fusion) and inter-specific CAT fusions (after 96 h), the fused conidia (*n* = 40) were inoculated on PDA plates and allowed to grow as a sporulating colony individually. From these sporulating colonies, single conidia were isolated by following single spore isolation method (Ho and Ko 1997) and again inoculated on PDA plates. The pure colonies originating from single spore isolation were considered as putative heterokaryotic/homokaryotic progenies. The colony morphologies of these putative heterokaryotic/homokaryotic progenies were analyzed. In cases of inter-specific CAT fusion, the species backgrounds of the putative heterokaryotic progenies were checked by *ApMAT* gene sequencing so as to find out whether they belong to *C. gloeosporioides* or *C. siamense*. The generated *ApMAT* gene sequences were deposited in NCBI-GenBank. In order to rule out the possibility of any other mechanisms (e.g. spontaneous phenotypic heterogeneity or stress induced genomic alterations) for generation of phenotypic diversity, the single conidium of each species were harvested from the 17 days old cultures of these two fungi, grown separately and without subjecting them to CAT fusion. These vegetatively grown conidia were allowed to develop as a colony and their morphological characteristics were also recorded.

### Amplified fragment length polymorphism (AFLP) analysis of *C. gloeosporioides* and *C. siamense* parental strains and their putative heterokaryotic progenies

In order to determine genetic diversity in putative heterokaryotic progenies generated post inter-specific CAT fusion between *C. gloeosporioides* and *C. siamense*, an AFLP assay was performed for *C. gloeosporioides* parent strain (CGP), *C. siamense* parent strain (CSP), *C. gloeosporioides* heterokaryotic progenies (CG1–10) and *C. siamense* heterokaryotic progenies (CS1–10) by following a previously described AFLP protocol (Chakrabarti et al. 2010). The native polyacrylamide gel electrophoresis (nPAGE) of AFLP PCR products were run on C-DASG-400-50 Dual Adjustable Mega-Gel Electrophoresis System (CBS Scientific) at 200 Volts for 3–4 h. After the electrophoresis, the gel was stained with Silver Nitrate stain (Elouafi and Nachit 2004). The stained gel was observed under white light and photographed with Canon EOS 600D.

### Assessment of hybrid vigour of heterokaryotic and homokaryotic progenies under stress conditions

The fitness of parent strains and putative heterokaryotic progenies of *C. gloeosporioides* generated post inter-specific CAT fusion between *C. gloeosporioides* and *C. siamense* was tested under different stresses viz. oxidative stress (H_2_O_2_), osmotic stresses (NaCl and Sorbitol) by employing surface plate assay (Zafra et al. 2015). As a control experiment, fitness of homokaryotic progenies generated post intra-specific (self) CAT fusion in *C. gloeosporioides* were also assessed. Briefly, the *C. gloeosporioides* heterokaryotic and homokaryotic progenies and parent strain (1 × 10^4^ conidia) were individually inoculated on the centre of PDA plates containing 30 mM H_2_O_2_, 1 M NaCl and 1 M Sorbitol separately. Plates were incubated at 25 °C for 7 days and mycelium radial extension rate measurements (cm d^− 1^) were made every 24 h manually with help of ruler. Fungal growth assays were structured by using an 11 × 3 factorial design (11 strains and 3 different stress media).

### Statistical analyses

All data were analyzed by one-way Analysis of Variance (ANOVA) with Tukey’s multiple comparison post-hoc test using GraphPad Prism 5 Statistics Software. Differences with a *p-value* < 0.05 were considered statistically significant. All assays were performed in triplicates (3 replicate per sample) and 150 conidial pairs were counted per replicate, wherever applicable.

## RESULTS

### High frequency of inter-specific versus intra-specific CAT fusion in *C. gloeosporioides* and *C. siamense*

**Fig. 1:**
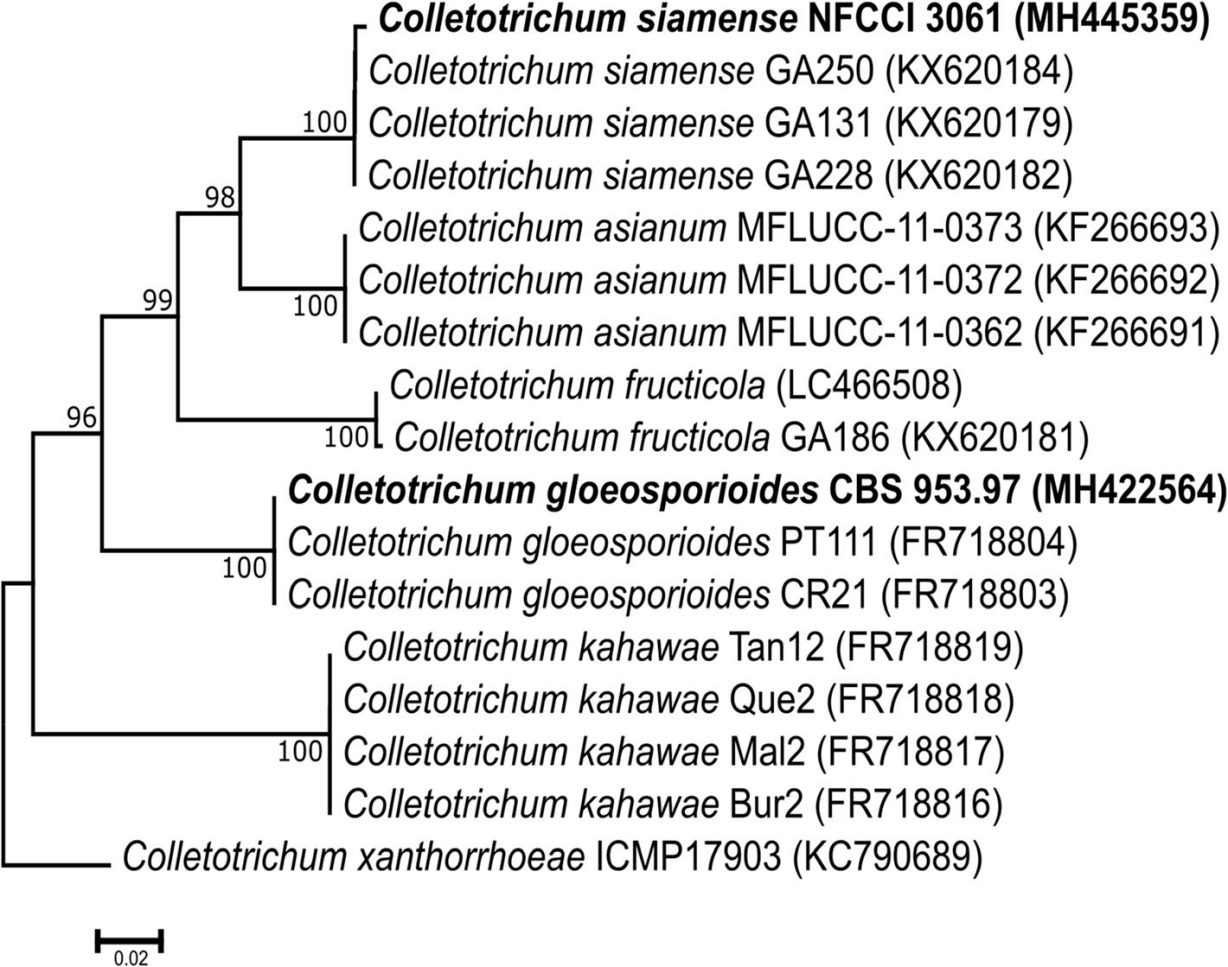
*ApMAT* gene-based phylogenetic placement of *C. gloeosporioides* (CBS 953.97) and *C. siamense* (NFCCI 3061) with the closely related species. The tree was constructed using the neighbor joining method in MEGA 7. The scale bar indicates the number of expected substitutions per site. The numbers provided on branches are frequencies with which a given branch appeared in 1000 bootstrap replications. The tree was rooted with *C. xanthorrhoeae*

**Fig. 2:**
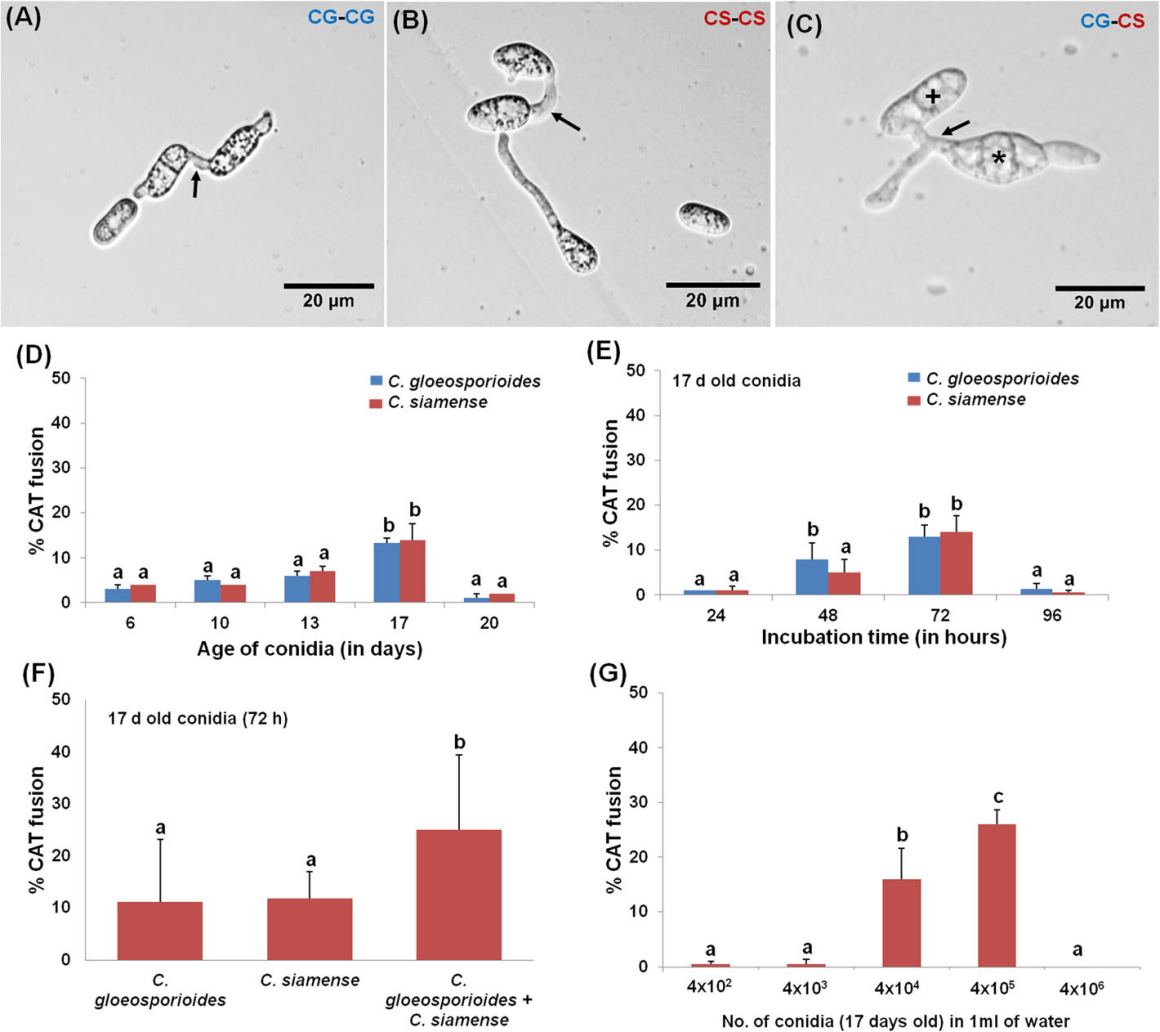
Intra and inter-specific CAT fusion in water in *C. gloeosporioides* and *C. siamense*. **a** Intra-specific CAT fusion in 17 days old *C. gloeosporioides* conidia. **b** Intra-specific CAT fusion in 17 days old *C. siamense* conidia. **c** Inter-specific CAT fusion between 17 days old *C. gloeosporioides* and *C. siamense* conidia. **d** Percentage CAT fusion in different aged conidia (4 × 10^5^ in 1 ml of water) viz. 6, 10, 13, 17 and 20 days of *C. gloeosporioides* and *C. siamense* 72 h post-incubation. **e** Percentage CAT fusion in 17 days old conidia (4 × 10^5^ in 1 ml of water) of *C. gloeosporioides* and *C. siamense* incubated for different incubation time viz. 24, 48, 72 and 96 h. **f** Percentage CAT fusion in 17 days old conidia (4 × 10^5^ in 1 ml of water) of *C. gloeosporioides* and *C. siamense* grown and incubated individually and in co-culture in water for 72 h. **g** Percentage CAT fusion in different conidial densities viz. 4 × 10^2^, 4 × 10^3^, 4 × 10^4^, 4 × 10^5^ and 4 × 10^6^ of 17 days old conidia incubated in 1 ml of water for 72 h. Arrow indicates CAT fusion, asterisk (*) symbol indicates *C. siamense* conidia and plus (+) symbol indicates conidia of *C. gloeosporioides*. Cg: *C. gloeosporioides* and Cs: *C. siamense.* CAT fusion was quantified as the percentage of conidia involved in fusion. Average from 3 replicates (*n* = 3) and 150 conidial pairs were counted per replicate. Bar indicates standard deviation. Statistical significance of differences was analyzed by one-way ANOVA with Tukey’s multiple comparison post-hoc test (bars with the same letter are not significantly different; *p* ≤ 0.05). Scale Bar = 20 μm

The BLASTn analysis of *ApMAT* DNA sequence of *C. gloeosporioides* CBS 953.97 and *C. siamense* NFCCI 3061 showed 100% sequence similarity with *C. gloeosporioides* and *C. siamense,* respectively (Table S1). Further, the *ApMAT* based phylogenetic analysis revealed that these two species of *Colletotrichum* clustered in their respective clades in phylogenetic tree, thereby confirming their identity and phylogenetic positions (Fig. 1). *Colletotrichum gloeosporioides* could also be differentiated from *C. siamense* by having cylindrical conidia while the later had fusiform shaped conidia (Fig. 2A-C) (Prihastuti et al. 2009). When different aged conidia of *C. gloeosporioides* and *C. siamense* were analyzed individually for CAT fusion, it was observed that the CAT fusion occurred in very low frequency in 6 days old conidia and thereafter percentage of CAT fusion increased with the increasing age of conidia. The CAT fusion frequency reaches a statistically significant peak when the conidial age was 17 days (ANOVA + Tukey’s post-hoc test, *n* = 3, *p* < 0.05) (Fig. 2D). It was observed that when 17 days old conidia of *C. gloeosporioides* and *C. siamense* were incubated in distilled water for 24, 48, 72 and 96 h separately, the maximum CAT fusion frequency was observed at 72 h post-incubation (ANOVA + Tukey’s post-hoc test, *n* = 3, *p* < 0.05) (Fig. 2E). The CAT fusion percentage observed in *C. gloeosporioides* and *C. siamense* individually (intra-specific) were 11% ± 2 and 12% ± 3.6%, respectively (Fig. 2F). However, when 17 days old conidia of these two different species were co-cultured in distilled water for CAT induction (for 72 h), interestingly the inter-specific CAT fusion percentage observed was as high as 25% ± 5%, which was significantly higher than intra-specific CAT fusion percentage (Fig. 2F).

The representative microscopic images of intra-specific and inter-specific CAT fusion in/between *C. gloeosporioides* and *C. siamense* have also been depicted in Fig. 2A-C. Based on these results, further experiments were conducted using the optimized parameters of 17 days old conidia and 72 h incubation time.

**Fig. 3:**
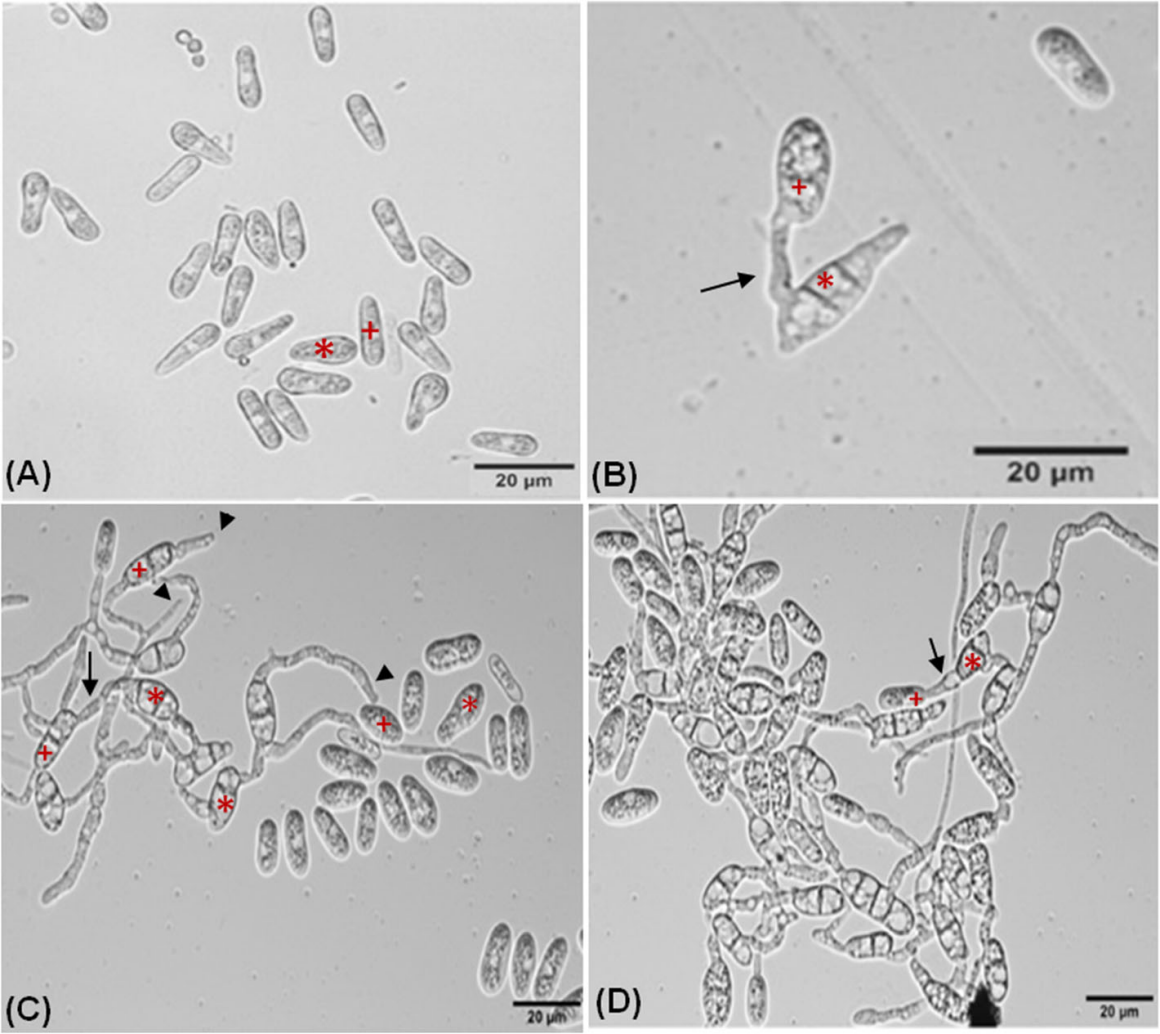
Different stages of inter-specific CAT fusion between *C. gloeosporioides* and *C. siamense* in-vitro in water. **a** CAT induction. **b** CAT fusion, **c** CAT homing and CAT fusion. **d** CAT network. Arrow indicates CAT fusion, arrowhead indicates CAT homing, asterisk (*) symbol indicates *C. siamense* conidia and plus (+) symbol indicates conidia of *C. gloeosporioides*. Scale Bar = 20 μm

Different stages of the inter-specific CAT fusion between *C. gloeosporioides* and *C. siamense* were studied (Fig. 3). We hypothesized that, initially conidia get primed for CAT fusion due to the effect of secretary QSMs, constituting the first stage of CAT fusion known as CAT induction (Fig. 3a). Subsequently, CATs home towards each other and finally they undergo fusion (Fig. 3b, c). Later on, with increasing incubation time CAT connections expanded and formed CAT network (Fig. 3d).

When younger conidia (6 days old) were incubated in 100% PDB, they exhibit significant GT formation and no CAT fusion at all. When the same younger conidia were incubated in water, they could not form GT at all and could undergo CAT fusion to some extent (10.7% ± 5%) (Fig. S2). On the contrary, when the older conidia (17 days old) were incubated in 100% PDB, no GT formation was observed while when they were incubated in water, the conidia could efficiently undergo CAT fusion (25% ± 4%) (Fig. S2). As the age of conidia increases the GT formation percentage decreases, on the contrary, the CAT fusion percentage increased with the increasing age of conidia (Fig. S2). This experiment indicates that the GT and CAT are mutually exclusive processes.

### CAT fusion is dependent on nutrient availability

**Fig. 4:**
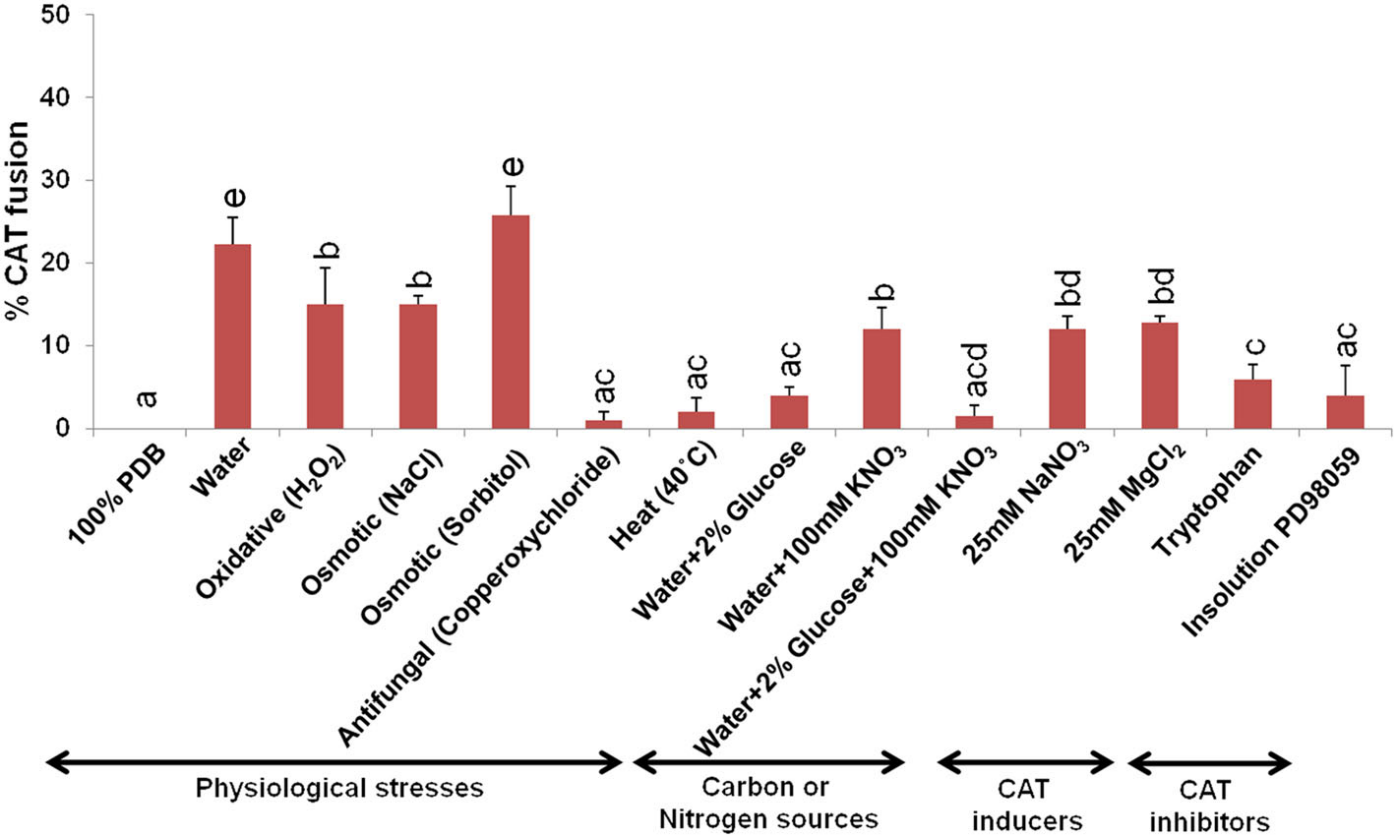
Effects of physiological stresses, nutrients, CAT inducers and CAT inhibitors on inter-specific CAT fusion frequency in *C. gloeosporioides* and *C. siamense*. Inter-specific CAT fusion percentage in water, PDB, oxidative stress (H_2_O_2_), osmotic stress (NaCl and sorbitol), heat, antifungal stress, glucose, KNO_3_, combination of glucose and KNO_3_, NaNO_3_, MgCl_2_, tryptophan, Insolution™ PD98059 (inhibitor of MAPKK pathway). Average from 3 replicates (*n* = 3) and 150 conidial pairs were counted per replicate. Bar indicates standard deviation. Statistical significance of differences was analyzed by one-way ANOVA with Tukey’s multiple comparison post-hoc test (bars with the same letter are not significantly different; *p* ≤ 0.05)

The conidia of *C. gloeosporioides* and *C. siamense* failed to undergo CAT fusion in the presence of nutrient rich medium like 100% PDB (Fig. 4). We have observed a significant CAT fusion frequency (25% ± 5%) in water (result “High frequency of inter-specific versus intra-specific CAT fusion in *C. gloeosporioides* and *C. siamense*” section). However, when the conidia were incubated in water with glucose, KNO_3_ and combination of glucose and KNO_3_, the CAT fusion frequency decreased to 4% ± 1, 12% ± 2.6 and 2% ± 1.3%, respectively (Fig. 4). Among different stress conditions, under osmotic (1 M NaCl and 1 M sorbitol) and oxidative stresses (30 mM H_2_O_2_) high frequency of CAT fusion was observed in these fungi (Fig. 4). The maximum CAT fusion (25.7% ± 4.3%) was detected under osmotic stress exerted by 1 M sorbitol, which was even greater than water (Fig. 4). The inter-specific CAT fusion was also observed in the presence of other known CAT inducers like 25 mM NaNO_3_, and 25 mM MgCl_2_ at the tune of 12% ± 1.5, and 12.8% ± 1% respectively, however, the percentage CAT fusion was lower than water (Fig. 4). Presence of tryptophan could reduce the CAT fusion to 5.9% ± 1.7%. The conidia failed to induce CAT fusion in the presence of InSolution™ PD 98059, an inhibitor of MAP kinase kinase, thereby suggesting the involvement of a MAPK pathway in inter-specific CAT fusion between *C. gloeosporioides* and *C. siamense* (ANOVA + Tukey’s post-hoc test, *n* = 3, *p* < 0.05) (Fig. 4).

### Inter-specific CAT fusion is mediated through quorum sensing like phenomenon

**Fig. 5:**
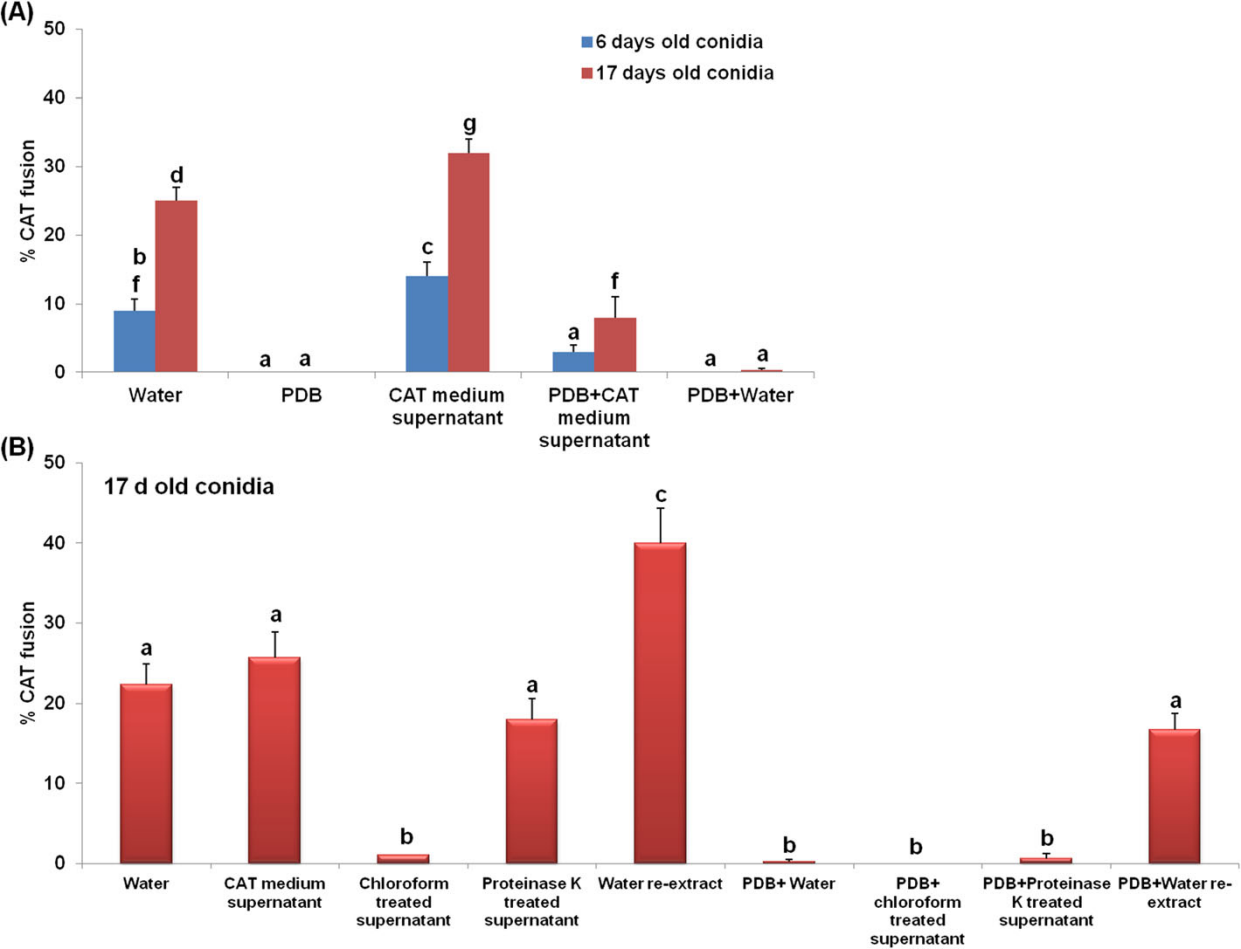
Inter-specific CAT fusion in *C. gloeosporioides* and *C. siamense* is mediated through quorum sensing phenomena. **a** Percentage CAT fusion in young (6 days) and old (17 days) conidia in water, PDB, CAT medium supernatant, PDB + CAT medium supernatant and PDB + water. **b** Percentage CAT fusion in 17 days old conidia in water, CAT medium supernatant, chloroform treated CAT medium supernatant, proteinase K treated CAT medium supernatant, water re-extract of chloroform treated CAT medium supernatant, PDB + water, PDB+ chloroform treated CAT medium supernatant, PDB + proteinase K treated CAT medium supernatant, PDB + water re-extract of chloroform treated CAT medium supernatant. Water was originally used to generate the CAT medium supernatant. Average from 3 replicates (*n* = 3) and 150 conidial pairs were counted per replicate. Bar indicates standard deviation. Statistical significance of differences was analyzed by one-way ANOVA with Tukey’s multiple comparison post-hoc test (bars with the same letter are not significantly different; *p* ≤ 0.05). Scale Bar = 20 μm

A density of 4 × 10^4^ and 4 × 10^5^ conidia per ml of water was found to be optimal for inter-specific CAT fusion (Fig. 2G), while the maximum CAT fusion percentage (26% ± 2.6%) was seen in 4 × 10^5^ conidia per ml (ANOVA + Tukey’s post-hoc test, *n* = 3, *p* < 0.05) (Fig. 2G). When inter-specific CAT fusion in young (6 days) and old (17 days) conidia of both the species were assessed in water, the younger conidia showed low levels of CAT fusion (11% ± 0.8%) as compared to the older conidia (23% ± 1%). While in the presence of 100% PDB, conidia of both ages showed zero CAT fusion, thereby suggesting that rich nutrients do not support CAT fusion. Interestingly, an increase in CAT fusion percentage was seen in the presence of CAT medium supernatant (14% ± 1% in 6 days and 27% ± 1% in 17 days old conidia), suggesting that the QSMs present in CAT medium supernatant could even induce CAT fusion in younger conidia. A statistically significant increase in the percentage of CAT fusion was seen in both young (8% ± 0.5%) and old conidia (14% ± 1.7%) when grown in 100% PDB supplemented with CAT medium supernatant (ANOVA + Tukey’s post-hoc test, *n* = 3, *p* < 0.05) (Fig. 5A). When PDB was diluted with water in the same proportion as the PDB + CAT supernatant treatment, we did not observe any CAT fusion. This indicates that increased CAT fusion was not due to dilution of PDB in PDB + CAT supernatant treatment (Fig. 5A). The proteinase K treated CAT medium supernatant could induce inter-specific CAT fusion (18% ± 2.6%), while chloroform treated CAT medium supernatant failed to induce CAT fusion in these fungi, suggesting that the putative QSM or CAT inducer was extractable in chloroform (Fig. 5B). The leftover chloroform from chloroform treatment of CAT medium supernatant was re-extracted with water and interestingly such water re-extract could induce a significantly high CAT fusion percentage (up to 40% ± 4.3%) (ANOVA + Tukey’s post-hoc test, *n* = 3, *p* < 0.05), and thereby suggesting that the CAT inducer was extractable in chloroform and then water (Fig. 5B). Chloroform treated, and proteinase K treated chemical fractions were not able to induce CAT fusion in the presence of equal volume of PDB. Another chemical fraction consisting of PDB and water re-extract could induce CAT fusion up to some extent (16.6% ± 2%). These results indicate that CAT fusion was dependent on conidial density and putative extracellular QSMs.

### Inter-specific CAT fusion involve movement of nuclei and other cell organelles

**Fig. 6:**
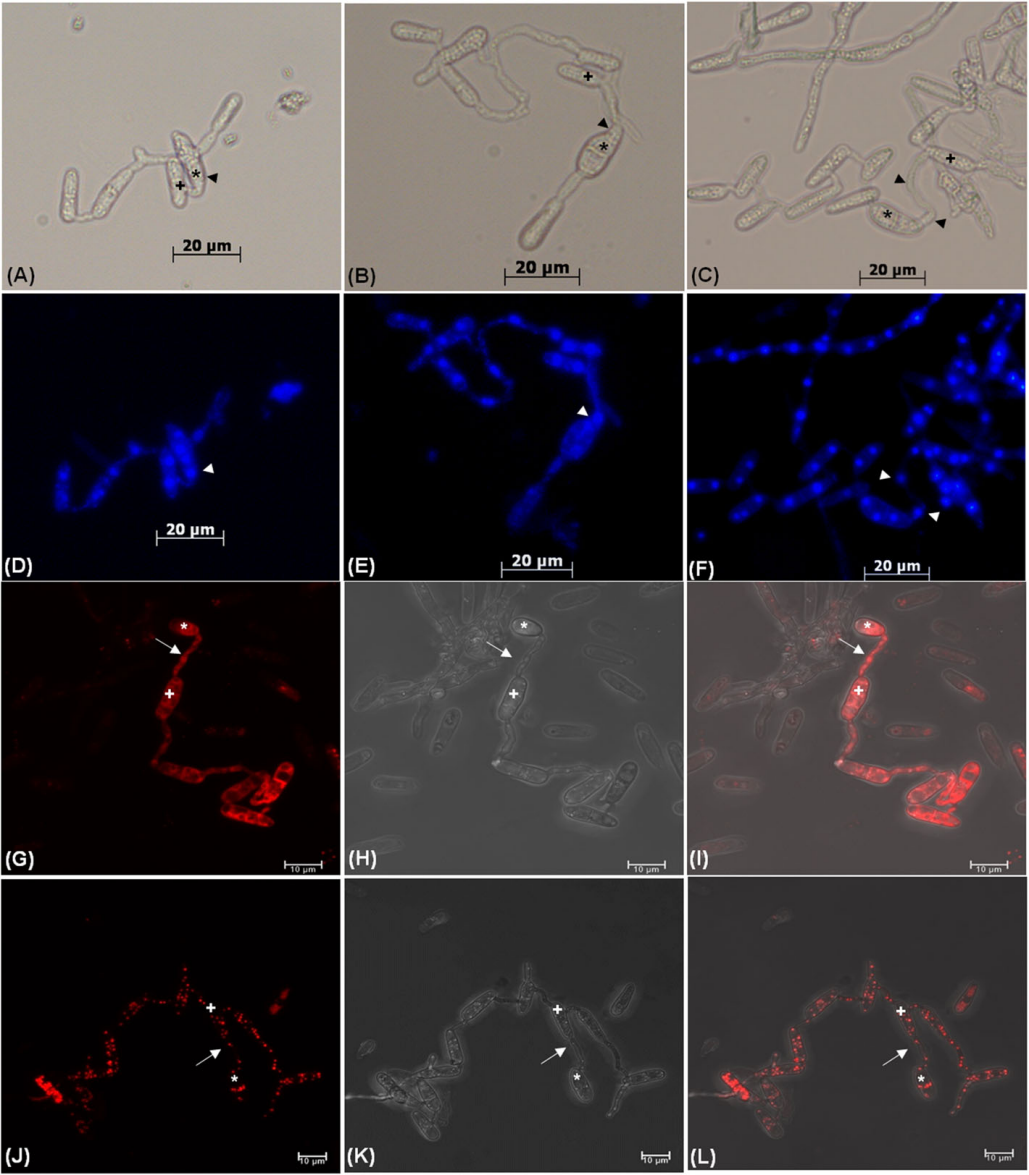
Transfer of nucleus and different cell organelles during CAT fusion between *C. gloeosporioides* and *C. siamense*. **a**, **b** and **c** Bright-field microscopy of CAT fusion between the 17 days old conidia *C. gloeosporioides* and *C. siamense*. **d**, **e** and **f** Visualization of nuclear transfer between the 17 days old conidia of *C. gloeosporioides* and *C. siamense* by DAPI staining using fluorescence microscope. **g** Fluorescent, **h** Brightfield and **i** Overlapping microscopic images of mitochondrial movement by MitoRed staining. **j** Fluorescent, **k** Brightfield and **l** Overlapping microscopic images of movement of lipid droplets by Nile red staining. Arrowhead indicates nucleus, arrow indicates CAT fusion, asterisk (*) symbol indicates *C. siamense* conidia and plus (+) symbol indicates conidia of *C. gloeosporioides*. Scale bar = 20 μm for images (**a**-**f**) and 10 μm for images (**g**-**l**)

DAPI staining showed that approximately 80% of the conidia of both species were bi-nucleated before CAT fusion, however, the number of nuclei increased up to 3–4 in the beginning of nuclear transfer between the conidia during CAT fusion. Out of such 3–4 nuclei, one nucleus/part of nucleus was transferred through CATs between conidia of these two species (Fig. 6A-F). Mito Red staining revealed the movement of mitochondria through fused CATs between conidia of *C. gloeosporioides* and *C. siamense* (Fig. 6G-I). The transfer of lipid droplets through CATs was also evident by Nile Red staining (Fig. 6J-L).

### Inter-specific CAT fusion generated phenotypic and genotypic diversity

**Fig. 7:**
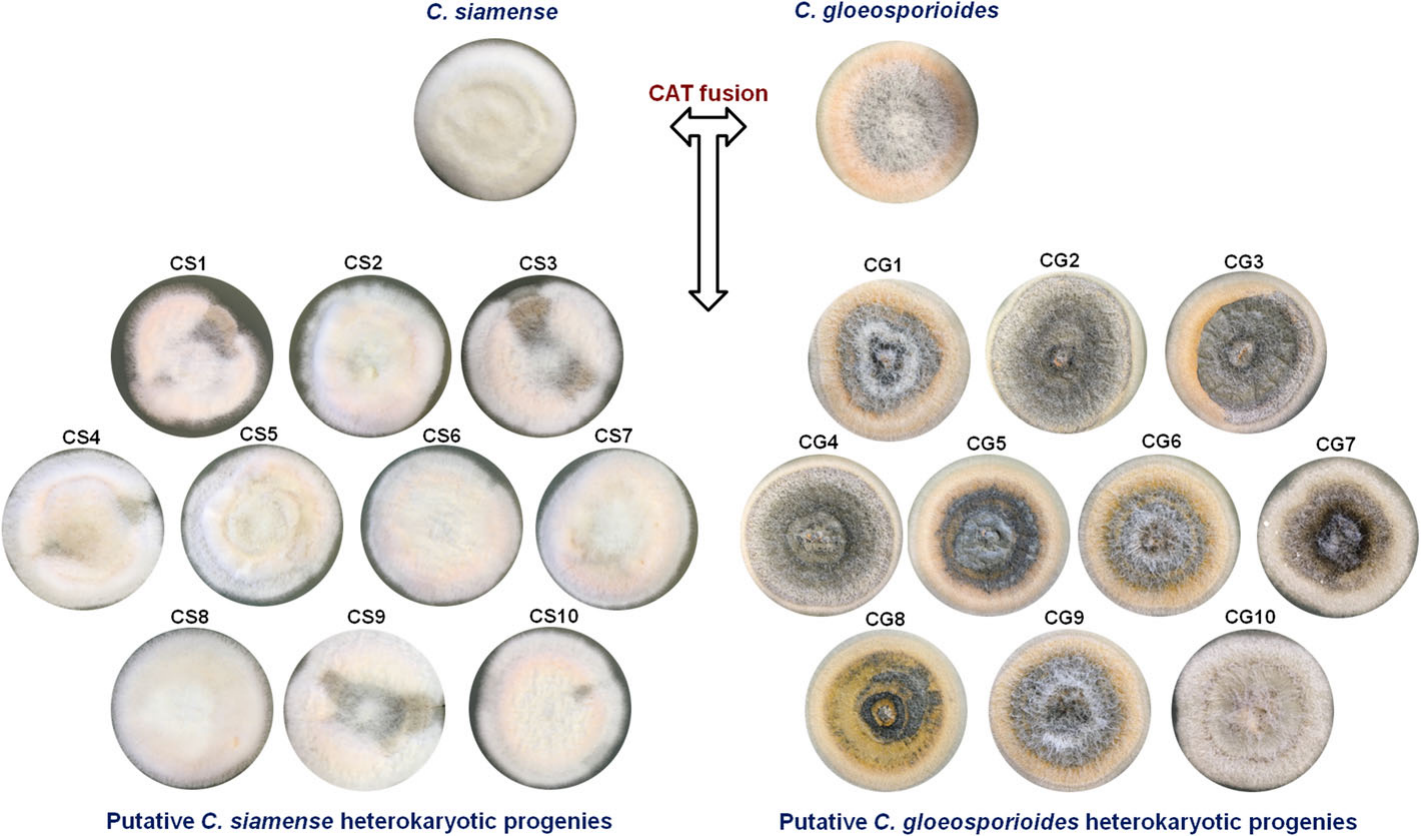
Colony morphology of *C. gloeosporioides* and *C. siamense* parent strains and their post-CAT fusion progenies. Colony morphology of parent *C. gloeosporioides* and *C. siamense* strains and their putative heterokaryotic progenies CG1-CG10 and CS1-CS10, respectively derived by single spore isolation from a mixture of conidia that had undergone CAT fusion

The putative heterokaryotic progenies generated through inter-specific CAT fusion between *C. gloeosporioides* and *C. siamense* showed phenotypic variations in colony characteristics as compared to their parent strains (Fig. 7)*.* We selected 10 such putative heterokaryotic progenies of each *C. gloeosporioides* (CG1 to CG10) and *C. siamense* (CS1 to CS10) species background (Fig. 7). Among both the species background, a greater number of *C. gloeosporioides* putative heterokaryotic progenies showed significant phenotypic variations as compare to *C. siamense* putative heterokaryotic progenies (Table S2). Microscopic analysis of the parent strains and the putative heterokaryotic progenies of *C. gloeosporioides* and *C. siamense* revealed that they were indeed pure cultures only. Further, the *ApMAT* gene sequencing of these putative heterokaryotic progenies accurately identified their species background, thereby ruling out the possibility of conidial mixing of these two species. The *ApMAT* gene sequences generated in this study were deposited in GenBank with accession numbers listed in Table S1.

Possibility of any other mechanisms for generation of phenotypic diversity e.g. intra-specific CAT fusion (or self-CAT fusion), spontaneous phenotypic heterogeneity or stress induced genomic alterations were ruled out, because progenies generated through intra-specific CAT fusion (or self-CAT fusion) in *C. gloeosporioides* and *C. siamense* individually did not show any apparent phenotypic variations in colony morphology (Fig. S3A). Further, the single conidia (17 days old) of each species when grown vegetatively as colony (without CAT fusion), they did not show any phenotypic variations, thereby suggesting that the phenotypic variations obtained were due to a possible transfer of genetic material through inter-specific CAT fusion only (Fig. S3B).

**Fig. 8:**
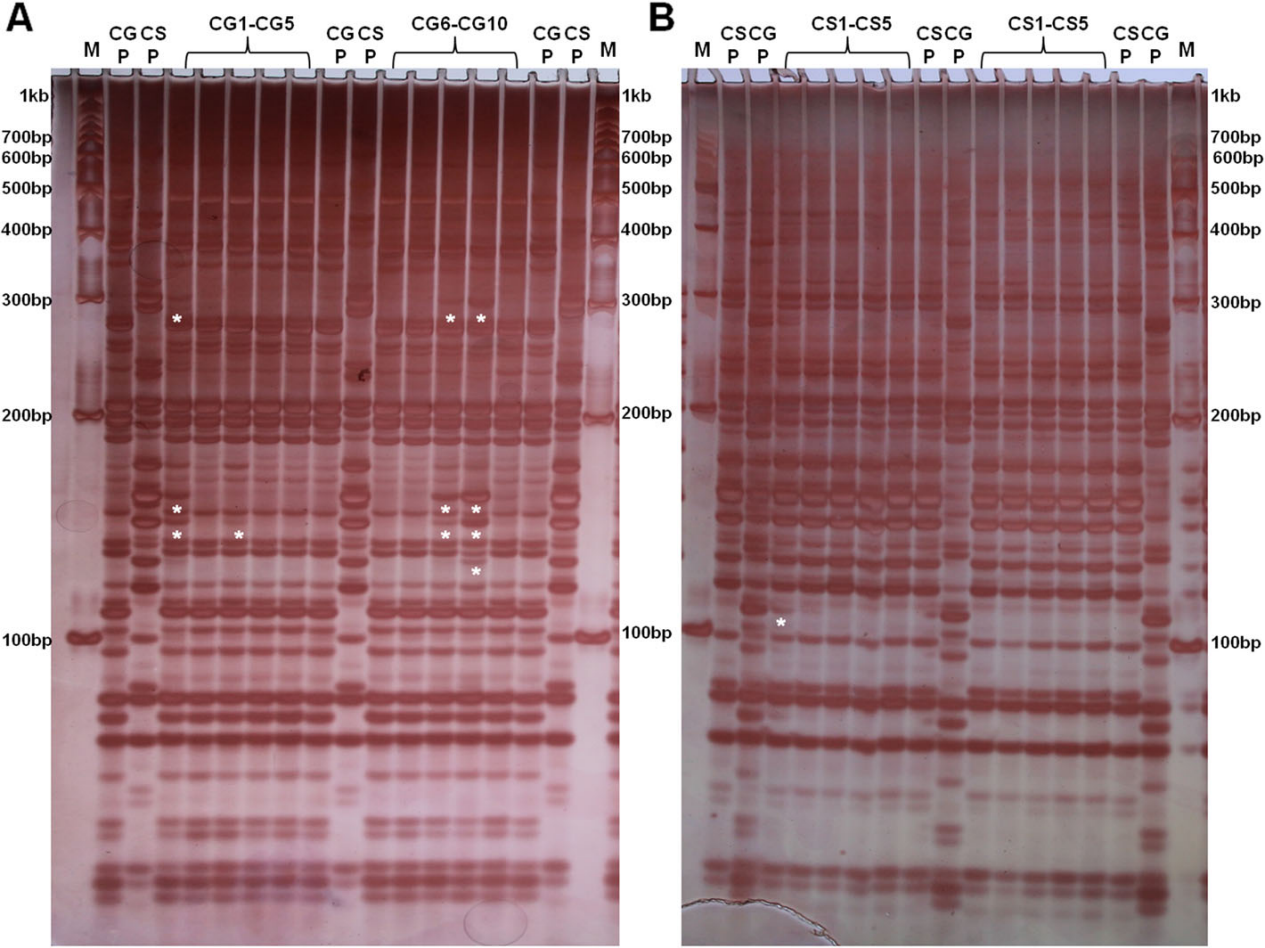
AFLP analysis of *C. gloeosporioides* and *C. siamense* parent strains and their putative heterokaryotic progenies. **a** AFLP banding patterns of parent *C. gloeosporioides* (CGP), *C. siamense* (CSP) and putative heterokaryotic progenies of *C. gloeosporioides* (CG1-CG10). **b** AFLP banding patterns of parent *C. siamense* (CSP), *C. gloeosporioides* (CGP) and putative heterokaryotic progenies of *C. siamense* (CS1-CS10) along with 100 bp DNA marker (M). Asterisk indicates the presence of extra bands beneath them

The AFLP banding pattern of some heterokaryotic progenies of *C. gloeosporioides* and *C. siamense* were different compared to their parent strains. We observed significantly more variations in AFLP banding pattern in *C. gloeosporioides* heterokaryotic progenies namely CG1, 3, 8 and 9 as compared to *C. gloeosporioides* parent strain (Fig. 8A). On the other hand, very fewer variations were observed in *C. siamense* heterokaryotic progenies banding pattern (only in CS1) as compared to *C. siamense* parent strain (Fig. 8B). The presence of extra band was indicated as an asterisk just beneath them in Fig. 8. Interestingly, the extra bands observed in *C. gloeosporioides* heterokaryotic progenies CG1, 3, 8 and 9 was not present in *C. gloeosporioides* parent strain but present in *C. siamense* parent strain (Fig. 8A), which denotes that these extra bands in *C. gloeosporioides* heterokaryotic progenies might have got transferred from *C. siamense* through CAT fusion, thereby suggesting an inter-specific genetic transfer.

### Putative heterokaryotic progenies showed varied fitness under stress conditions

**Fig. 9:**
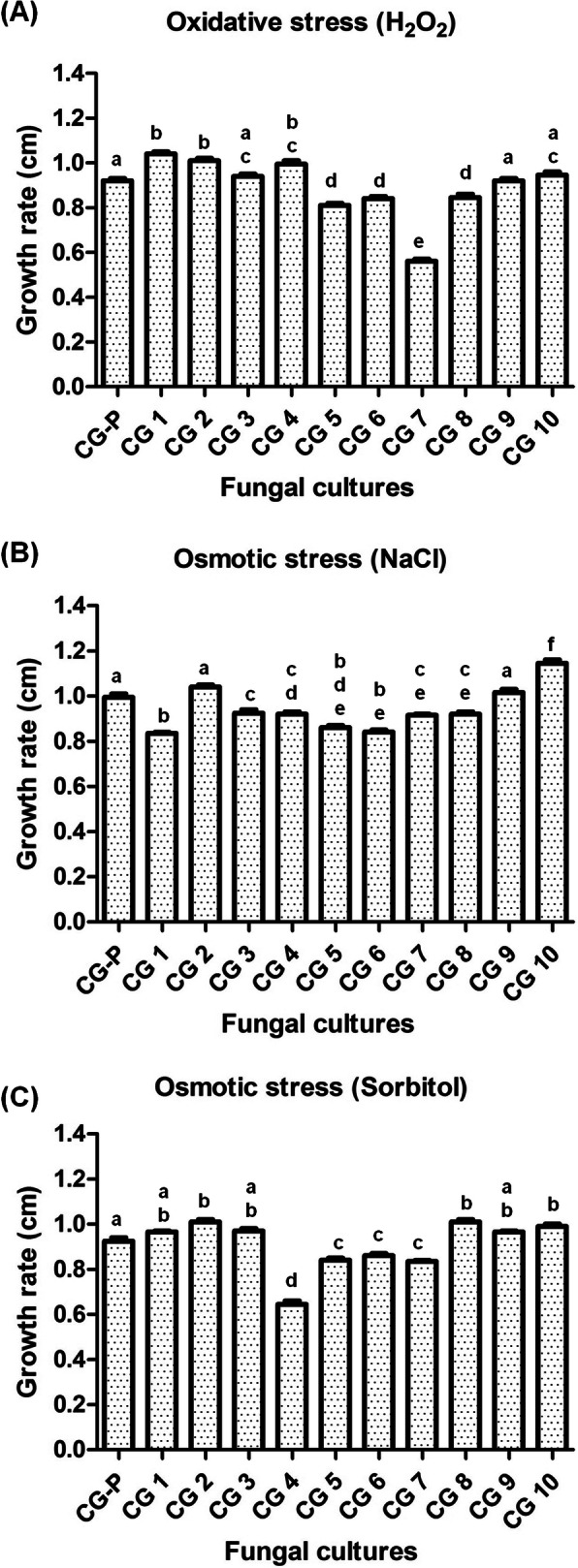
Growth rate (cm) of *C. gloeosporioides* parent (CGP) and their heterokaryotic progenies (CG1–10) under different stresses. **a** Growth rate in presence of oxidative stress induced by H_2_O_2_. **b** Growth rate in presence of osmotic stress induced by NaCl. **c** Growth rate in osmotic stress induced by sorbitol. Average from 3 replicates and bar indicates standard deviation. Statistical significance of differences was analyzed by one-way Tukey’s multiple comparison post-hoc test (bars with the same letter are not significantly different; *p* ≤ 0.05)

Out of 10 *C. gloeosporioides* putative heterokaryotic progenies, 7 showed significant growth rate variation under oxidative stress, out of which three heterokaryotic progenies showed higher growth rate as compared to the parent strain. On the contrary, 4 out of 7 heterokaryotic progenies showed reduced growth rate under oxidative stress (Fig. 9A). With respect to osmotic stress (1 M NaCl), only 1 out of 10 putative heterokaryotic progenies showed higher growth rate, and 7 showed reduced growth rate as compared to the parent strain (Fig. 9B). Further under sorbitol mediated osmotic stress, 3 out of 10 putative heterokaryotic progenies showed higher growth rate, and 4 showed reduced growth rate as compared to the parent strain (ANOVA + Tukey’s post-hoc test, *n* = 3, *p* < 0.05) (Fig. 9C). In another control experiment, wherein, we assessed the growth rates of homokaryotic progenies under oxidative and osmotic stresses, we did not observe any significant variations in their growth rates (ANOVA + Tukey’s post-hoc test, *n* = 3, *p* < 0.05) (Fig. S4A-C).

## DISCUSSION

The studied fungal species *C. gloeosporioides* and *C. siamense* have previously been shown to be different valid species, however, the *ApMAT* based phylogenetic analysis has shown that *C. gloeosporioides* was more closely related to *C. siamense* then *C. asianum* and *C. fructicola*. (Silva et al. 2012; Sharma et al. 2013). Our strains belonging to these two species were also rightly placed in the phylogenetic tree (Fig. 1). We have optimized the in-vitro conditions for CAT induction in *C. gloeosporioides* and *C. siamense.* We observed significant CAT induction in 17 days old conidia of both the species individually (Fig. 2D). Among different species of *Colletotrichum*, it was reported that 16 days old culture of *C. lindemuthianum* and *C. gossypii* undergo CAT fusion, while 20 days old culture of *C. fructicola* and *C. nymphaeae* formed CATs (Gonçalves et al. 2016; Roca et al. 2003, 2004). If CAT fusion in other fungal genera is considered, the conidial age requirements for *F. oxysporum* and *N. crassa* were 7–10 and 4–5 days, respectively (Kurian et al. 2018; Roca et al. 2005a; Shahi et al. 2016). Our result seems to fit with the conidial age requirement (older conidia) of the genus *Colletotrichum* for CAT induction. The CAT fusion percentage was about 11% ± 3.6% for both *C. gloeosporioides* and *C. siamense* individually (Fig. 2F). However, the CAT fusion frequency was significantly high (25%) when these two species were co-cultured for CAT induction, suggesting that the inter-specific CAT fusion was preferred as compare to intra-specific CAT fusion in these two fungal species (Fig. 2F). Inter-specific CAT fusion has also been demonstrated between *C. lindemuthianum* and *C. gossypii,* however, the frequency of these inter-specific fusions was very low even on strong hygromycin selection (Roca et al. 2004). Interestingly, in our experiments, there was no selection pressure for inter-specific CAT fusion between *C. gloeosporioides* and *C. siamense,* still the CAT fusion with the other species was preferred. It is hypothesized that inter-specific CAT fusion provides a unique opportunity to exchange the gene pool between two different species, which would possibly generate more genetic diversity as compared to intra-specific CAT fusion.

It has been reported previously that the older conidia were found to be optimal for CAT fusion while the younger conidia were optimal for GT formation (Ishikawa et al. 2010). We have tested the abilities of different aged conidia to form GT and CAT and have shown that the CAT fusion occurs only in water and it gets inhibited in 100% PDB while GT formation occurs in 100% PDB but not in water (Fig. S2). As the age of conidia increases the GT formation percentage decreases in PDB and CAT fusion frequency increases in water. Our results also indicate that the older conidia were superlative for CAT fusion while younger conidia were ideal for GT formation.

While studying the effect of availability of nutrients on CAT fusion frequency, we observed that the conidia of *C. gloeosporioides* and *C. siamense* failed to undergo CAT fusion in the presence of 100% PDB (Fig. 4). Even the presence of glucose, KNO_3_, and combination of glucose and KNO_3_, further lessen the CAT fusion frequency as compared to water (Fig. 4). These observations suggest that the CAT fusion in *C. gloeosporioides* and *C. siamense* occur only in nutrient limited conditions. Our observation corroborates with the previous findings in several ascomycetes’ fungi, wherein, the CAT fusion was inhibited in the presence of nutrients or rich organic growth medium (Roca et al. 2005a; Ishikawa et al. 2010; Shahi et al. 2016; Kurian et al. 2018). The known CAT inducers of *Fusarium sp.* e.g., NaNO_3_, and MgCl_2_ could induce some level of CAT fusion in *C. gloeosporioides* and *C. siamense*, however, the CAT fusion frequency was significantly less as compared to water suggesting that the physiological requirement for CAT induction in different fungi may not be very identical.

A correlation has been established between some important developmental processes like sporulation and biofilm formation to the environmental stresses in few ascomycetous fungal species (Emri et al. 2015; Orosz et al. 2018; Zheng et al. 2015). Therefore, when the effect of physiological stresses on CAT fusion frequency was tested, we observed that the CAT fusion was found to be higher in oxidative and osmotic stresses (Fig. 4). Even the hyperosmotic stress exerted by sorbitol induced much higher CAT fusion percentage as compared to water (Fig. 4). The possible reason for relatively high frequency CAT fusion by hyperosmotic and oxidative stresses may be because these stresses activate the mitogen-activated protein kinase signaling pathway, which was shown to be essential for CAT fusion in *N. crassa* (Roca et al. 2005a). A chemical agent InSolution™ PD98059 has previously been shown to inhibit MEK in *C. gloeosporioides* (Kim et al. 2000). We have shown that the inter-specific CAT fusion in *C. gloeosporioides* and *C. siamense* gets inhibited by a MEK inhibitor InSolution™ PD 98059 (Fig. 4), thereby suggesting a possible role of MAP kinase pathway in CAT fusion in these fungi. Two MAPK pathways are essential for communication and cell fusion in *N. crassa*: the cell wall integrity/MAK-1 pathway and the MAK-2 (signal response) pathway. Previous studies have demonstrated several points of cross-talk between the MAK-1 and MAK-2 pathways, which is likely necessary for coordinating chemotropic growth toward an extracellular signal, and then mediating cell fusion (Fischer et al. 2018; Fischer and Glass 2019; Fleissner et al. 2009; Teichert et al. 2014). Therefore, we hypothesize that a potential cross-talking among MAPK cascades during hyperosmotic and oxidative stresses, in turn induce CAT fusion fortuitously, however, further studies are needed in this direction.

In *C. lindemuthianum, C. gossypii, C. fructicola, C. nymphaeae, F. oxysporum, N. crassa* and *V. inaequalis*, the CAT induction is dependent on conidial density which was more or less similar i.e. 1 × 10^6^ conidia/ml (Gonçalves et al. 2016; Kurian et al. 2018; Leu 1967; Roca et al. 2003, 2005a; Shahi et al. 2016). The combined conidial number requirement for inter-specific CAT fusion in *C. gloeosporioides* and *C. siamense* was also found to be 4x10^5^conidia/ml indicating the CAT fusion is mediated through quorum sensing (Fig. 2G). The chemical identity of extracellular QSM is not known to date in any fungi responsible for CAT fusion (Roca et al. 2005a). We have shown that the young conidia were not optimal for CAT fusion, however, when the young conidia were incubated in filtered CAT medium supernatant of older conidia (17 days), they could also undergo CAT fusion up to some extent, thereby suggesting that some molecule/s are secreted in the medium, which could induce CAT fusion even in the younger conidia (Fig. 5A). We have also demonstrated that presence of nutrients (100% PDB) inhibited CAT fusion in *C. gloeosporioides* and *C. siamense,* however, when the conidia of these fungi were incubated in 100% PDB supplemented with CAT medium supernatant, then the conidia of both the age could undergo CAT fusion, thereby, suggesting that though the nutrients (100% PDB) had the potential to inhibit the CAT fusion but the QSMs could still induce the CAT fusion in the presence of nutrients (Fig. 5A). It can be argued that this increase in CAT fusion percentage even in the presence of PDB might be due to the dilution of PDB, however, in another control experiment, we have demonstrated that PDB diluted with water could not induce CAT fusion (Fig. 5A). It was suggested that the CAT inducer/ QSMs might be of peptide or proteinaceous in nature, which gives species specificity for CAT fusion (Roca et al. 2005a). We found that proteinase K treated CAT medium supernatant could retain the CAT inducing ability, thereby indicating that the potential QSMs was not a peptide or protein. However, the chloroform extraction abolishes the CAT induction ability of the CAT medium supernatant, suggesting that the potential CAT inducer was extractable in chloroform (Fig. 5B). Further, we have also shown that when leftover chloroform from chloroform treatment of CAT medium supernatant was re-extracted with water and such re-extract could induce significantly high CAT fusion percentage (up to 40% ± 4.3%) in older (17 days) conidia (Fig. 5B). The high CAT fusion percentage seen in re-extract could be because the QSM got purified from other impurities, thereby increased the CAT fusion percentage. In a different set of experiments, we have shown that when the chloroform and proteinase K treated fractions were mixed with equal volume of PDB, they could not induce CAT fusion in these fungi. However, putative QSMs in water re-extract (apparently more purified form) could still induce CAT fusion in presence of PDB (Fig. 5B), which corroborates with our results, wherein, we have shown some extent of CAT fusion using CAT medium supernatant in presence of PDB (Fig. 5A). We are currently trying to decipher the chemical structure and identity of this QSM.

Previous studies have indicated that CAT fusion may facilitate horizontal gene/chromosome transferred. Nuclei were shown to move between fused conidia of many fungi viz. *C. lindemuthianum, C. gossypii, C. nymphaeae, F. oxysporum,* and *N. crassa* (Gonçalves et al. 2016; Kurian et al. 2018; Roca et al. 2003, 2005a; Shahi et al. 2016). We also observed the movement of nuclei during CAT fusion between *C. gloeosporioides* and *C. siamense*, which signifies that this inter-specific CAT fusion is not accidental; indeed, it does involve exchange of nuclear material (Fig. 6). However, with this result, it is difficult to comment whether the recombination or nuclear fusion had occurred or not, therefore this state of having nuclei from two different species together could be considered as heterokaryotic state. In *C. lindemuthianum*, true nuclear fusion was demonstrated in 27% of the heterokaryotic cells generated by CAT fusion (Ishikawa et al. 2012). Therefore, tagging *C. gloeosporioides* and *C. siamense* with different fluorescent markers would also allow us to follow the nuclear dynamics (including nuclear fusion) in future. Movement of other cell organelles e.g., mitochondria, and lipid droplets were also shown during the CAT fusion, which signifies that there has been an active exchange of cellular content between the two species (Fig. 6).

Nuclear transfer through CAT fusion could be a form of  HGT resulting in either heterokaryosis or recombination, which in turn might generate phenotypic and genetic diversity. It has been reported that the inter-specific CAT fusion between *C. lindemuthianum* and *C. gossypii,* resulted some hybrid colonies, which exhibited morphological variation with distinct phenotypic sectors of both parental types (Roca et al. 2004). Occurrence of heterokaryotic sectored colonies post CAT fusion has also been demonstrated in *C. lindemuthianum* (Ishikawa et al. 2012)*.* We have also demonstrated that inter-specific CAT fusion between *C. gloeosporioides* and *C. siamense* generated heterokaryotic progenies with significant phenotypic variations in colony characteristics (Fig. 7, Table S2). By observing the colony characteristics, we could not really assign the species background. However, further identification was done by microscopic analysis of conidial shape and sizes. These two species could be differentiated based on their conidial morphologies, wherein, C*. gloeosporioides* conidia are cylindrical, while *C. siamense* are fusiform in shape (Prihastuti et al. 2009). Therefore, by doing light microscopic studies on these putative heterokaryotic progenies, we could tentatively establish the species background of these heterokaryons, however, at times it was difficult to differentiate the post CAT fusion generated conidia based on the morphology, therefore, the *ApMAT* gene sequencing accurately confirmed the species background of these putative heterokaryons (Table S1).

In order to prove that the phenotypic variations observed in putative heterokaryotic progenies were genuinely generated due to inter-specific CAT fusion between these two species, (1) the results of our control experiment showed that intra-specific CAT fusion (self-fusion) in *C. gloeosporioides* and *C. siamense* individually did not generate apparent colony morphology variations in their progenies (Fig. S3A). (2) To rule out the possibilities of phenotypic heterogeneity or stress induced genomic alterations (Coyle and Kroll 2008; Hewitt et al. 2016; Miousse et al. 2015) being the reasons of observed phenotypic variations, we have shown that when *C. gloeosporioides* and *C. siamense* were subjected to nutrient starvation, the colonies developed from resulting progenies did not show any significant phenotypic variation as compared to their parental strains (Fig. S3B). Therefore, the above-mentioned control experiments further confirmed that the phenotypic variation generated in putative heterokaryotic progenies was not spontaneous and was not the result of self-fusion.

We observed a much variable AFLP banding pattern in *C. gloeosporioides* putative heterokaryotic progenies as compared to *C. gloeosporioides* parent strain (Fig. 8). Even some extra bands of *C. siamense* origin were also detected in *C. gloeosporioides* heterokaryons. This indicates that during CAT fusion, some amount of DNA might have got transferred between the two species. These genotypic variations might be responsible for the above-mentioned phenotypic variations in *C. gloeosporioides* heterokaryotic progenies. Further, we do not see a significant variation in AFLP banding pattern in *C. siamense* heterokaryotic progenies, which interestingly corroborates with our phenotypic variation data, wherein, we did not see a significant phenotypic variation in the colonies of *C. siamense* heterokaryotic progenies as well. However, further experiments are warranted to confirm the genetic exchange between the species.

It has been speculated that in addition to hyphal fusion, the CAT fusion might also facilitate parasexual cycle via heterokaryosis in the genus *Colletotrichum* (da Silva et al. 2020). Among different species of *Colletotrichum*, a typical parasexual cycle consisting of a heterokaryon, diploids, and recombinants has been demonstrated in *C. lindemuthianum* (Rosada et al. 2010). While in *C. gloeosporioides*, heterokaryons were observed but neither diploid nor recombinants could be isolated and heterokaryosis was limited to the colony center (Chacko et al. 1994). Few of our *C. gloeosporioides* heterokaryotic progenies also showed phenotypic variations in the center of colony. Therefore, it is still not known whether, the heterokaryons are result of rapid haploidization of the diploid nucleus or it is just the transfer of DNA fragments? Further research needs to be done to elucidate the role of CAT fusion in parasexual cycle in *C. gloeosporioides.*

We have shown that a greater number of *C. gloeosporioides* heterokaryotic progenies exhibited phenotypic variations (Fig. 7, Table S2); therefore, when the fitness of these heterokaryons was tested, we observed that few heterokaryotic progenies (e.g. CG1 and CG4) showed growth advantages under certain stress conditions (oxidative stress) as compared to others and the same heterokaryons showed reduced growth under a different stress condition (osmotic stress). One putative *C. gloeosporioides* heterokaryotic progeny strain CG2 exhibited higher growth rates under oxidative as well as osmotic stress as compared to the parent strain of *C. gloeosporioides* (Fig. 9). There were no significant growth advantages or disadvantages observed in homokaryotic progenies generated post intra-specific CAT fusion under oxidative and osmotic stresses as compared to the parent strain of *C. gloeosporioides* (Fig. S4)*.* The observed growth advantages might be due to heterosis, wherein, the post inter-specific CAT fusion progenies were more heterozygous than their parents.

## CONCLUSION

This is the first study to determine the optimal conidial density, conidial age, nutritional factors, and other physiological requirements for CAT induction between *C. gloeosporioides* and *C. siamense*. We discovered a significantly efficient inter-specific CAT fusion between these two fungi under no selection pressure. Preliminary experimental proofs to link the role of inter-specific CAT fusion in generation of phenotypic and genotypic diversity in these fungi were also generated. The present study will help to further understand the genetic exchange/transfer mechanisms, parasexuality and generation of inter-specific hybrids or heterokaryons in *C. gloeosporioides* and *C. siamense* in future.

## Supplementary Information

**Additional file 1: Table S1.** Fungal cultures, their *ApMAT* gene-based identification and GenBank accession numbers.

**Additional file 2: Table S2.** Percentage phenotypic variations of *C. gloeosporioides* and *C. siamense* colonies generated post inter-specific CAT fusion.

**Additional file 3: Figure S1.** A schematic representation of in-vitro CAT induction protocol. Cg: *C. gloeosporioides* and Cs: *C. siamense*.

**Additional file 4: Figure S2.** Germ tube formation versus CAT fusion in *C. gloeosporioides* and *C. siamense.* a GT formation (in 100% PDB) and CAT fusion (in water) percentage in differentially aged conidia viz. 6, 10, 13 and 17 days. b A representative microscopic image of GT formation in 100% PDB. Average from 3 replicates (*n* = 3) and 150 conidial pairs were counted per replicate. Bar indicates standard deviation. Statistical significance of differences was analyzed by one-way ANOVA with Tukey’s multiple comparison post-hoc test (bars with the same letter are not significantly different; *p* ≤ 0.05). Scale Bar = 20 μm.

**Additional file 5: Figure S3.** Colony morphologies of *C. siamense* and *C. gloeosporioides* parent strains and their progenies generated post intra-specific CAT fusion and vegetative growth. a Colony morphologies of parent *C. siamense* and *C. gloeosporioides s*trains and their progenies (CSA-CSJ and CGA-CGJ) derived post intra-specific CAT fusion individually (self-fusion) by single spore isolation. b Colony morphologies of parent *C. siamense* and *C. gloeosporioides* strains and their progenies (CSV1-CSV10 and CGV1-CGV10) obtained from vegetatively grown *C. gloeosporioides* and *C. siamense* strains (17 days old)*,* individually without CAT fusion. Scale Bar = 20 μm.

**Additional file 6: Figure S4.** Growth rate (cm) of *C. gloeosporioides* parent (CGP), *C. siamense* parent (CGS) and their homokaryotic progenies (CGA-J) under different stresses. a Growth rate in presence of oxidative stress induced by H_2_O_2_. b Growth rate in presence of osmotic stress induced by NaCl. c Growth rate in osmotic stress induced by sorbitol. Average from 3 replicates and bar indicates standard deviation. Statistical significance of differences was analyzed by one-way Tukey’s multiple comparison post-hoc test (bars are not significantly different; p ≤ 0.05, hence different letters are not designated).

## Data Availability

*ApMAT* DNA Sequences of *C. gloeosporioides* and *C. siamense* parent and their heterokaryotic progeny strains were deposited to NCBI and accession numbers listed in Additional file 1: Table S1.

## Abbreviations

HGT: Horizontal gene transfer
HCT: Horizontal chromosome transfer
MAPK: Mitogen-activated protein kinase
GT: Germ tube
CAT: Conidial anastomosis tube
nPAGE: Native polyacrylamide gel electrophoresis
AFLP: Amplified fragment length polymorphism
CG: *Colletotrichum gloeosporioides*
CS: *Colletotrichum siamense*
RT: Room temperature
DIC: Differential interference contrast
NFCCI: National Fungal Culture Collection of India
MTCC: Microbial Type Culture Collection
h: Hour
QS: Quorum sensing
QSM: Quorum sensing molecule
MEK: MAP kinase kinase
