# Supplementary material for: Quorum sensing-mediated inter-specific conidial anastomosis tube fusion between Colletotrichum gloeosporioides and C. siamense
Source: IMA Fungus. 2021 Apr 1;12:7. doi: 10.1186/s43008-021-00058-y (PMC8015167; doi:10.1186/s43008-021-00058-y)
Supplement: Supplementary file 1 — Additional file 1: Table S1. Fungal cultures, their ApMAT gene-based identification and GenBank accession numbers. [file 43008_2021_58_MOESM1_ESM.docx]

**Supplementary table 1:** Fungal cultures, their *ApMAT* gene-based identification and GenBank accession numbers.

| **S. N.** | **Fungal strains** | **Code** | ***ApMAT* gene-based identification** | **GenBank accession numbers for *ApMAT* gene** |
| --- | --- | --- | --- | --- |
| 1 | *Colletotrichum gloeosporioides* parent strain | CG | *C. gloeosporioides* | MH422564 |
| 2 | Putative *C. gloeosporioides* heterokaryotic progenies (1–10) | CG1 | *C. gloeosporioides* | MH422565 |
|  |  | CG2 | *C. gloeosporioides* | MH445356 |
|  |  | CG3 | *C. gloeosporioides* | MH422566 |
|  |  | CG4 | *C. gloeosporioides* | MH422567 |
|  |  | CG5 | *C. gloeosporioides* | MH445357 |
|  |  | CG6 | *C. gloeosporioides* | MH422568 |
|  |  | CG7 | *C. gloeosporioides* | MH422569 |
|  |  | CG8 | *C. gloeosporioides* | MH422570 |
|  |  | CG9 | *C. gloeosporioides* | MH445358 |
|  |  | CG10 | *C. gloeosporioides* | MH422571 |
| 3 | *Colletotrichum siamense* parent strain | CS | *C. siamense* | MH445359 |
| 4 | Putative *C. siamense* heterokaryotic progenies (1–10) | CS1 | *C. siamense* | MH445360 |
|  |  | CS2 | *C. siamense* | MH445361 |
|  |  | CS3 | *C. siamense* | MH445362 |
|  |  | CS4 | *C. siamense* | MH445363 |
|  |  | CS5 | *C. siamense* | MH445364 |
|  |  | CS6 | *C. siamense* | MH445365 |
|  |  | CS7 | *C. siamense* | MH445366 |
|  |  | CS8 | *C. siamense* | MH445367 |
|  |  | CS9 | *C. siamense* | MH445368 |
|  |  | CS10 | *C. siamense* | MH445369 |
