# Supplementary material for: Quorum sensing-mediated inter-specific conidial anastomosis tube fusion between Colletotrichum gloeosporioides and C. siamense
Source: IMA Fungus. 2021 Apr 1;12:7. doi: 10.1186/s43008-021-00058-y (PMC8015167; doi:10.1186/s43008-021-00058-y)
Supplement: Supplementary file 2 — Additional file 2: Table S2. Percentage phenotypic variations of C. gloeosporioides and C. siamense colonies generated post inter-specific CAT fusion. [file 43008_2021_58_MOESM2_ESM.docx]

**Table S2:** Percentage phenotypic variations of *C. gloeosporioides* and *C. siamense* colonies generated post inter-specific CAT fusion.

| **Sr. No.** | **Fungal culture** | **Total number of fungal colonies isolated from single conidium post CAT fusion** | **Number of colonies with phenotypic variations** | **Percentage of colonies with phenotypic variations** | **Level of significance** |
| --- | --- | --- | --- | --- | --- |
| 1. | *C. gloeosporioides* | 20 | 9 | 45% | ** |
| 2. | *C. siamense* | 20 | 4 | 20% | ** |

** Indicates level of significance at *p-value* < 0.05 analyzed by unpaired t-test.
