# Supplementary figures and images for: Quorum sensing-mediated inter-specific conidial anastomosis tube fusion between Colletotrichum gloeosporioides and C. siamense
Source: IMA Fungus. 2021 Apr 1;12:7. doi: 10.1186/s43008-021-00058-y (PMC8015167; doi:10.1186/s43008-021-00058-y)

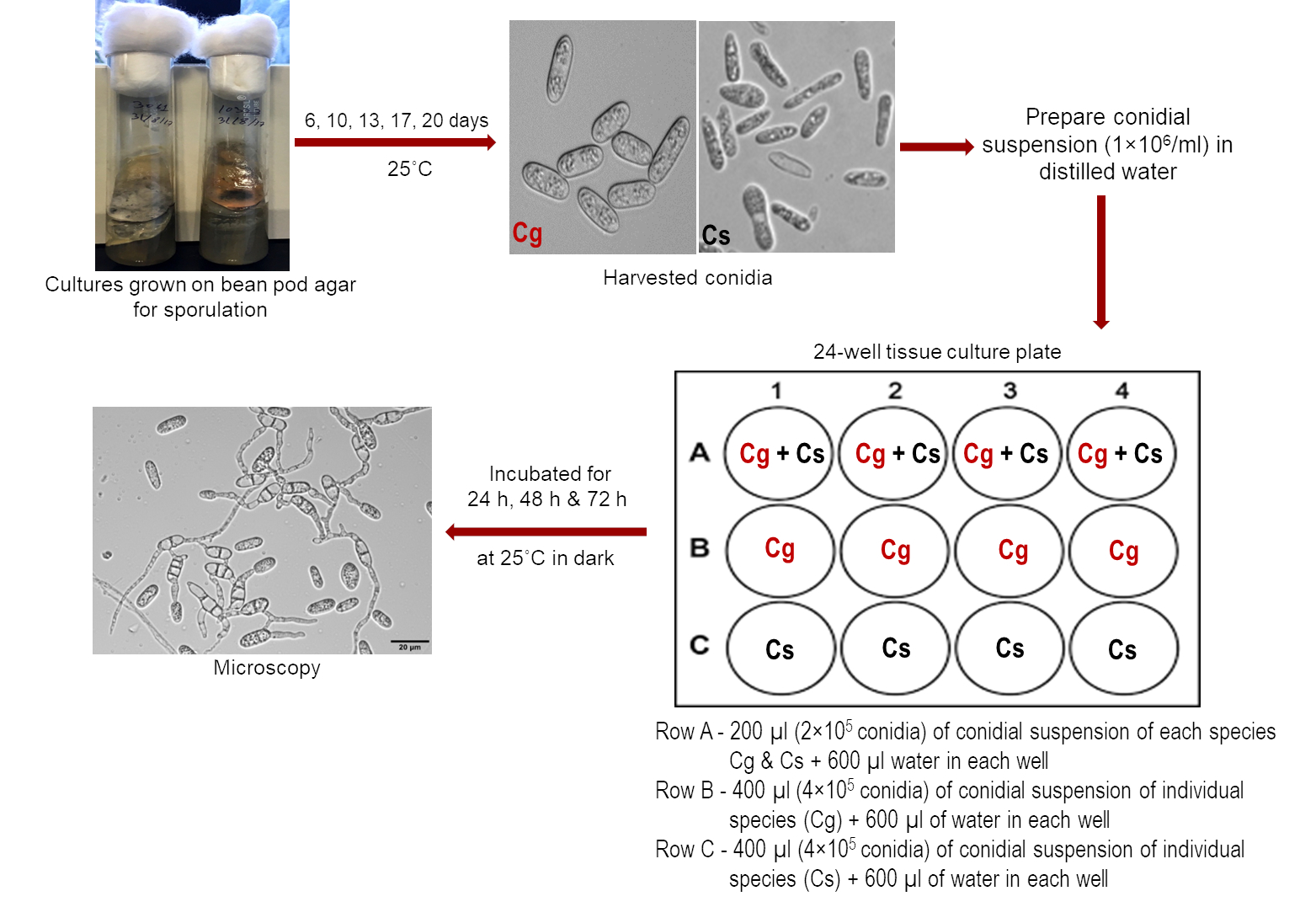

Supplement: Supplementary file 3 — Additional file 3: Figure S1. A schematic representation of in-vitro CAT induction protocol. Cg: C. gloeosporioides and Cs: C. siamense. [file 43008_2021_58_MOESM3_ESM.jpg]

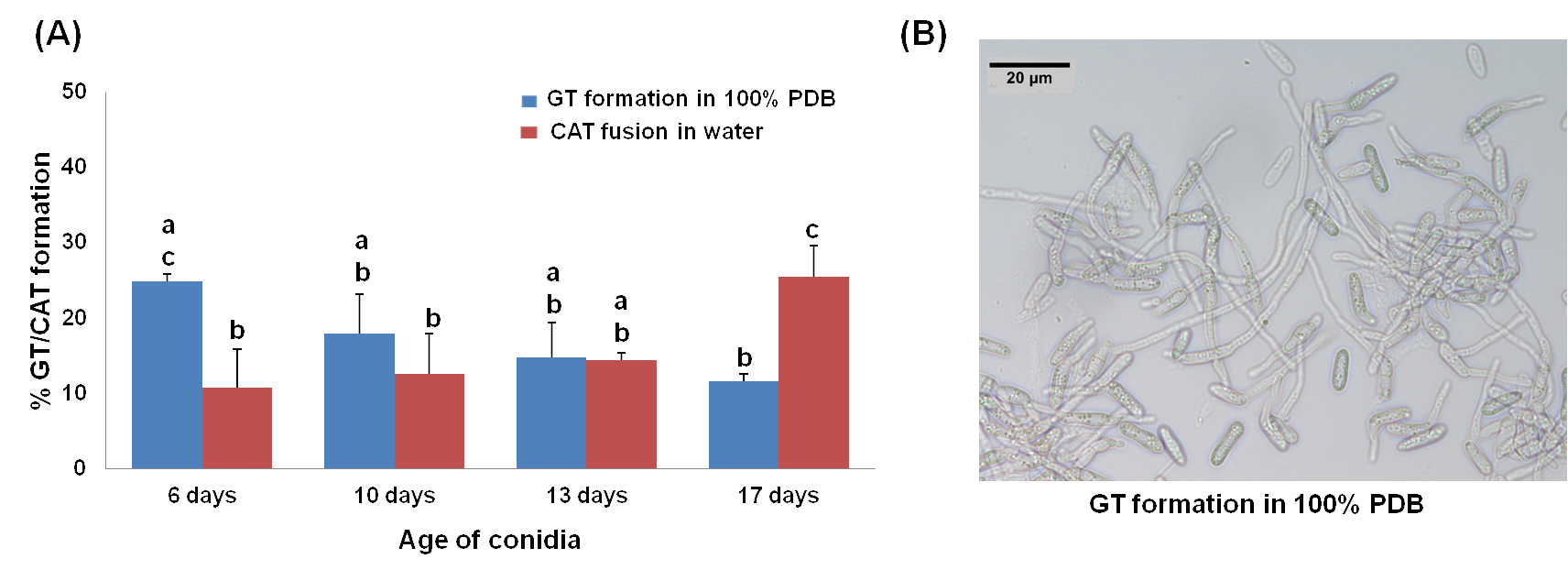

Supplement: Supplementary file 4 — Additional file 4: Figure S2. Germ tube formation versus CAT fusion in C. gloeosporioides and C. siamense. a GT formation (in 100% PDB) and CAT fusion (in water) percentage in differentially aged conidia viz. 6, 10, 13 and 17 days. b A representative microscopic image of GT formation in 100% PDB. Average from 3 replicates (n = 3) and 150 conidial pairs were counted per replicate. Bar indicates standard deviation. Statistical significance of differences was analyzed by one-way ANOVA with Tukey’s multiple comparison post-hoc test (bars with the same letter are not significantly different; p ≤ 0.05). Scale Bar = 20 μm. [file 43008_2021_58_MOESM4_ESM.jpg]

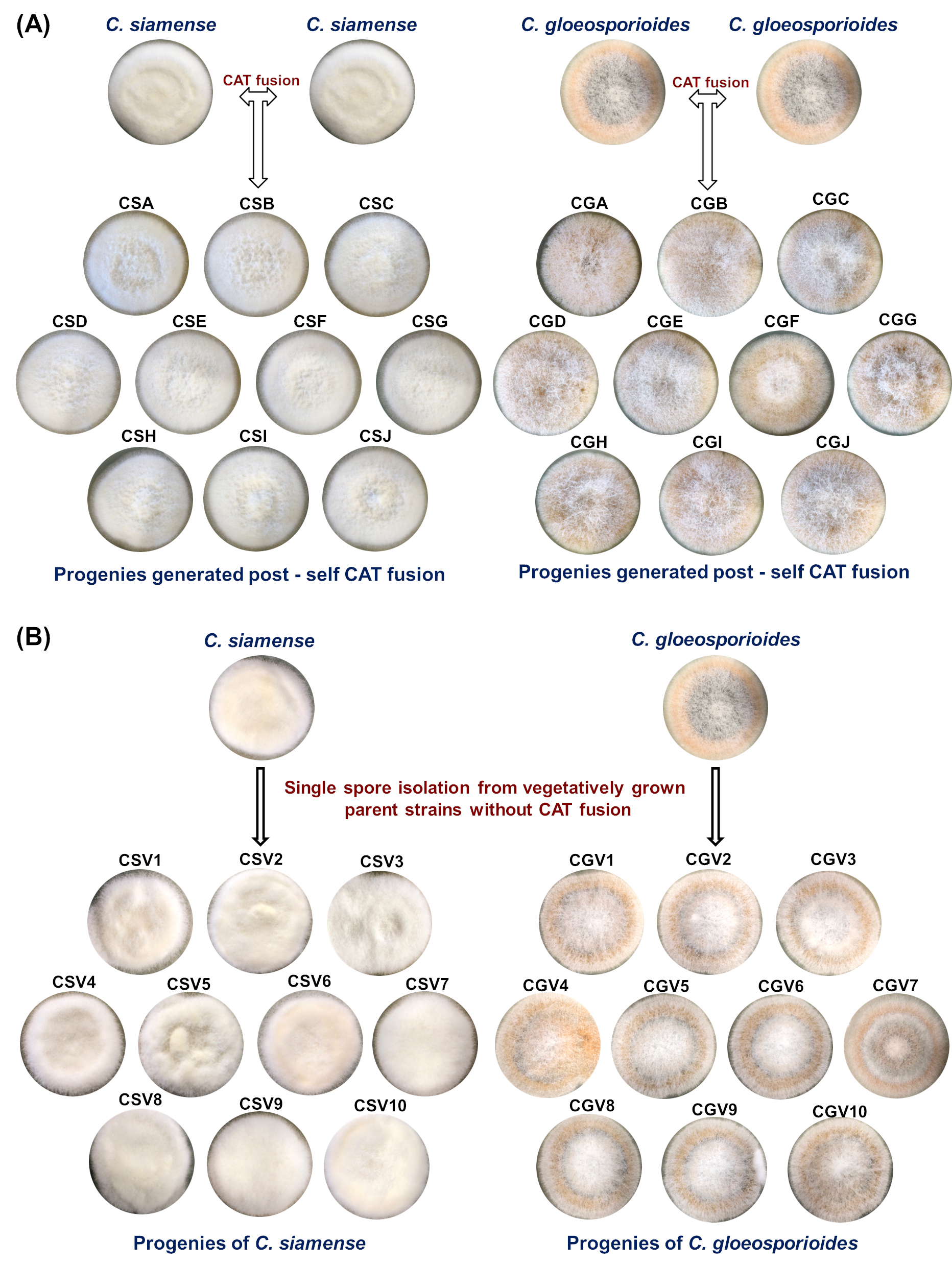

Supplement: Supplementary file 5 — Additional file 5: Figure S3. Colony morphologies of C. siamense and C. gloeosporioides parent strains and their progenies generated post intra-specific CAT fusion and vegetative growth. a Colony morphologies of parent C. siamense and C. gloeosporioides strains and their progenies (CSA-CSJ and CGA-CGJ) derived post intra-specific CAT fusion individually (self-fusion) by single spore isolation. b Colony morphologies of parent C. siamense and C. gloeosporioides strains and their progenies (CSV1-CSV10 and CGV1-CGV10) obtained from vegetatively grown C. gloeosporioides and C. siamense strains (17 days old), individually without CAT fusion. Scale Bar = 20 μm. [file 43008_2021_58_MOESM5_ESM.jpg]

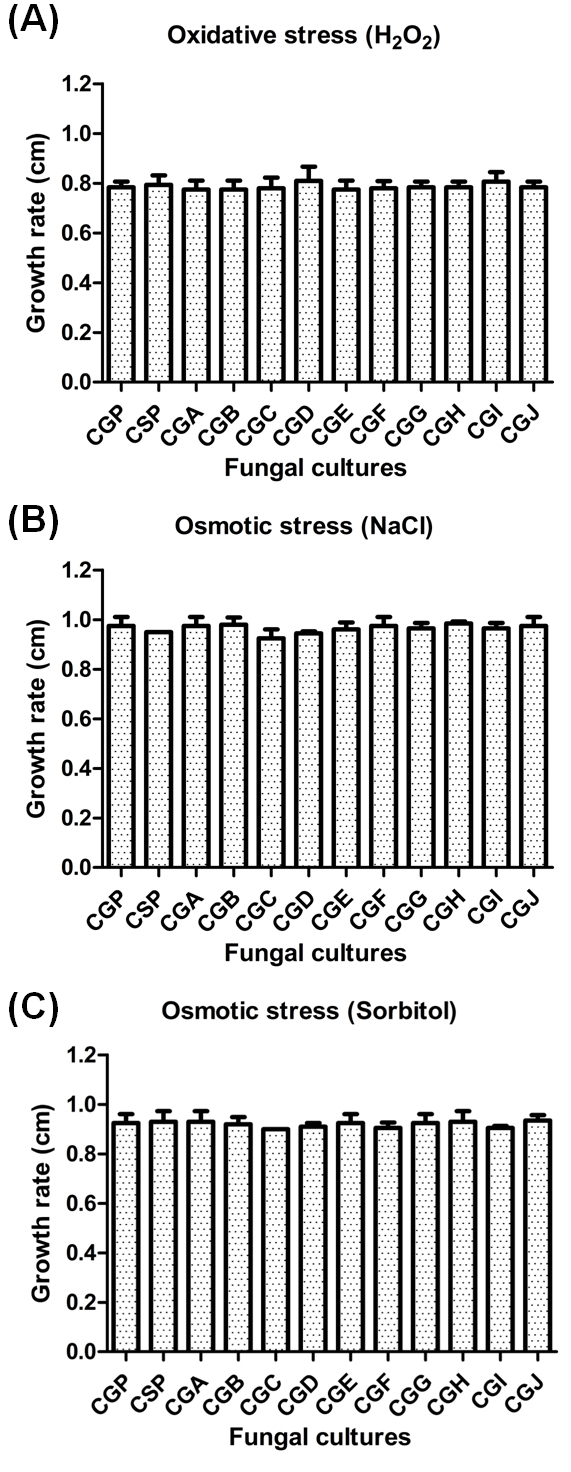

Supplement: Supplementary file 6 — Additional file 6: Figure S4. Growth rate (cm) of C. gloeosporioides parent (CGP), C. siamense parent (CGS) and their homokaryotic progenies (CGA-J) under different stresses. a Growth rate in presence of oxidative stress induced by H2O2. b Growth rate in presence of osmotic stress induced by NaCl. c Growth rate in osmotic stress induced by sorbitol. Average from 3 replicates and bar indicates standard deviation. Statistical significance of differences was analyzed by one-way Tukey’s multiple comparison post-hoc test (bars are not significantly different; p ≤ 0.05, hence different letters are not designated). [file 43008_2021_58_MOESM6_ESM.jpg]
